# Microglial mechanisms drive amyloid-β clearance in immunized patients with Alzheimer’s disease

**DOI:** 10.1038/s41591-025-03574-1

**Published:** 2025-03-06

**Authors:** Lynn van Olst, Brooke Simonton, Alex J Edwards, Anne V Forsyth, Jake Boles, Pouya Jamshidi, Thomas Watson, Nate Shepard, Talia Krainc, Benney Mr Argue, Ziyang Zhang, Joshua Kuruvilla, Lily Camp, Mengwei Li, Hang Xu, Jeanette L Norman, Joshua Cahan, Robert Vassar, Jinmiao Chen, Rudolph J Castellani, James Ar Nicoll, Delphine Boche, David Gate

**Affiliations:** 1 Abrams Research Center on Neurogenomics, Northwestern University Feinberg School of Medicine, Chicago, IL, USA.; 2 The Ken & Ruth Davee Department of Neurology, Northwestern University Feinberg School of Medicine, Chicago, IL, USA.; 3 Department of Pathology, Northwestern University Feinberg School of Medicine, Chicago, IL, USA.; 4 Bioinformatics Institute, Agency for Science, Technology and Research, Singapore, Singapore.; 5 Clinical Neurosciences, School of Clinical and Experimental Sciences, Faculty of Medicine, University of Southampton, Southampton, UK.; 6 Mesulam Center for Cognitive Neurology and Alzheimer’s Disease, Northwestern University Feinberg School of Medicine, Chicago, IL, USA.; 7 Centre for Computational Biology and Program in Cancer and Stem Cell Biology, Duke-NUS Medical School, Singapore, Singapore.; 8 Immunology Translational Research Program, Department of Microbiology and Immunology, Yong Loo Lin School of Medicine, National University of Singapore, Singapore, Singapore.; 9 Department of Cellular Pathology, University Hospital Southampton National Health Service Trust, Southampton, UK.; 10 Abrams Research Center on Neurogenomics, Northwestern University Feinberg School of Medicine, Chicago, IL, USA.; 11 The Ken & Ruth Davee Department of Neurology, Northwestern University Feinberg School of Medicine, Chicago, IL, USA.

## Abstract

Alzheimer’s disease (AD) therapies utilizing amyloid-β (Aβ) immunization have shown potential in clinical trials. Yet, the mechanisms driving Aβ clearance in the immunized AD brain remain unclear. Here, we use spatial transcriptomics to explore the effects of both active and passive Aβ immunization in the AD brain. We compare actively immunized patients with AD with nonimmunized patients with AD and neurologically healthy controls, identifying distinct microglial states associated with Aβ clearance. Using high-resolution spatial transcriptomics alongside single-cell RNA sequencing, we delve deeper into the transcriptional pathways involved in Aβ removal after lecanemab treatment. We uncover spatially distinct microglial responses that vary by brain region. Our analysis reveals upregulation of the triggering receptor expressed on myeloid cells 2 (TREM2) and apolipoprotein E (APOE) in microglia across immunization approaches, which correlate positively with antibody responses and Aβ removal. Furthermore, we show that complement signaling in brain myeloid cells contributes to Aβ clearance after immunization. These findings provide new insights into the transcriptional mechanisms orchestrating Aβ removal and shed light on the role of microglia in immune-mediated Aβ clearance. Importantly, our work uncovers potential molecular targets that could enhance Aβ-targeted immunotherapies, offering new avenues for developing more effective therapeutic strategies to combat AD.

## Main

For nearly three decades, clinical trials have targeted cerebral Aβ accumulation in AD^1^. Leading strategies include active and passive immunization against Aβ^2^. While these strategies can reduce cerebral Aβ^3–9^, they can also trigger adverse side effects^8–15^. Understanding the cellular mechanisms underpinning Aβ immunization is paramount to improving patient outcomes.

The AN1792 clinical trial was the first to actively immunize patients with AD^3^. This trial utilized immunization against a synthetic Aβ_1–42_ peptide. Preclinical studies showed promise, but the trial was suspended after some patients developed aseptic meningoencephalitis^10–12^ associated with cerebral amyloid angiopathy (CAA)^4,13^. Our prior postmortem analyses of AN1792 brains revealed Aβ clearance in some immunized patients, likely via microglia^3–5,16^. However, the microglial mechanisms dictating Aβ clearance in these brains remain unclear.

The inflammatory side effects from the AN1792 trial led to a shift toward passive immunization. In passive immunization, patients with AD receive antibodies that target Aβ, such as lecanemab. Lecanemab binds large soluble Aβ protofibrils, reduces Aβ markers and slows cognitive decline in early AD^9^. Our prior case study of a lecanemab-treated patient who developed stroke-like symptoms revealed inflammation in blood vessels with CAA and evidence of Aβ clearance^17,18^. Yet, the function of microglia in Aβ clearance in passive immunization also remains unclear.

In this study, we used spatial transcriptomics (ST) to analyze the neuroimmune response in AD brains following active and passive Aβ immunization. We compared AN1792-immunized Alzheimer’s disease (iAD) brains to nonimmunized Alzheimer’s disease (nAD) and control, non-neurologic disease (NND) brains. Additionally, we examined the neuroimmune response in the aforementioned patient treated with lecanemab using high-definition ST, spatial proteogenomics and single-cell RNA sequencing (scRNA-seq).

Our study uncovers distinct microglial phenotypes in Aβ-immunized AD brains and reveals genes that control clearance of Aβ by microglia. These results highlight candidate genes to modulate microglial responses in AD immunotherapy.

## Results

### Active Aβ immunization sustains inflammation at the Aβ niche

**Extended Data Fig. 1:**
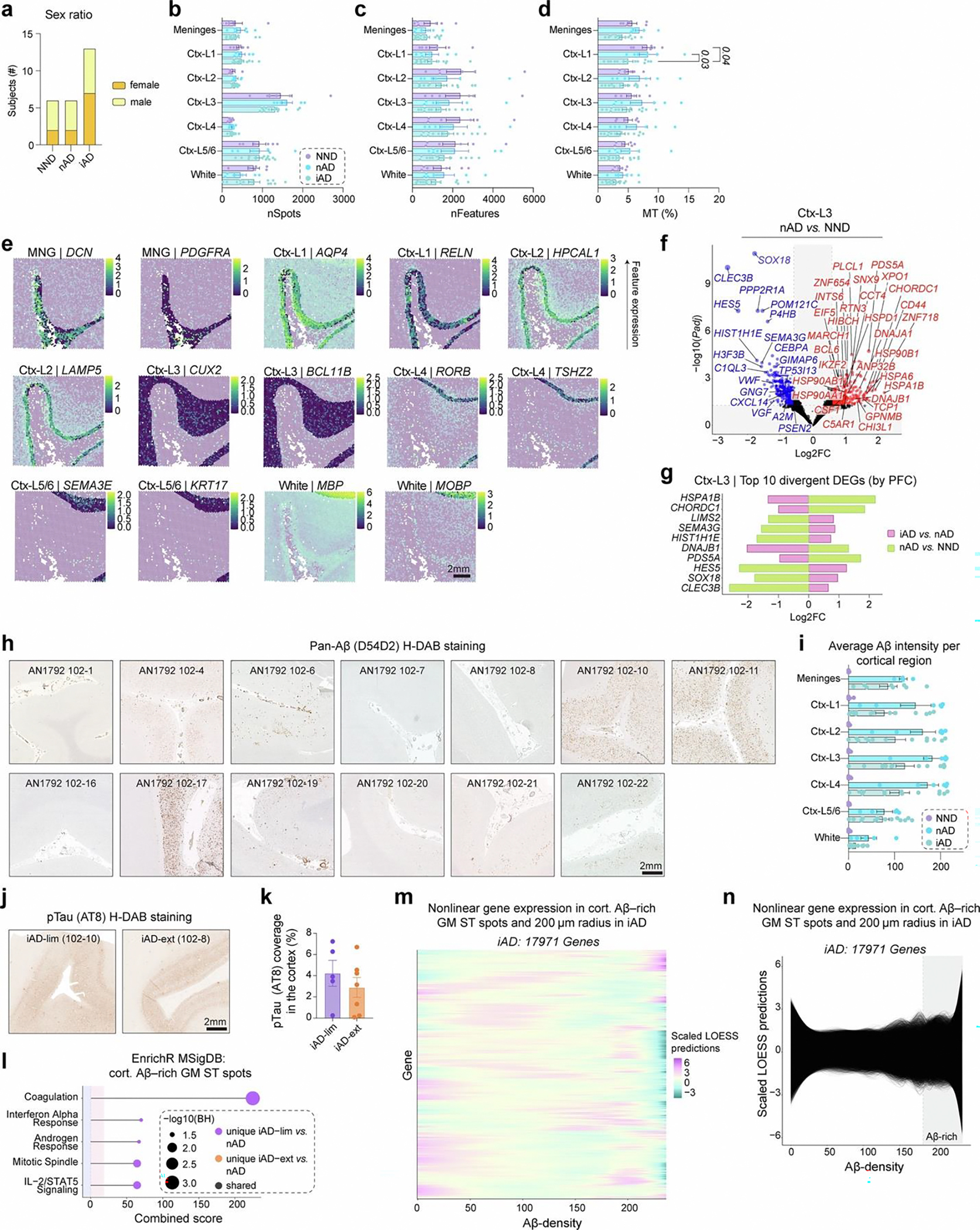
Active Aβ immunization sustains inflammation at the Aβ niche. **a**, Sex distribution per group**. b**, Number of ST spots per manually annotated area per donor for all groups**. c**, Average number of features (genes) per spot per manually annotated area per donor for all groups**. d**, Percentages of mitochondrial gene expression per spot averaged per manually annotated area per donor for all groups. **e**, Spatial plots showing expression of brain region-specific genes overlaid on corresponding manually annotated areas (shaded). **f**, Volcano plot of DEGs in cortical layer III (nAD vs. NND). **g**, Bar plot of the top 10 divergent DEGs in cortical layer III based on PFC, comparing iAD vs. nAD and nAD vs. NND. **h**, Pan-Aβ (D54D2) H-DAB staining for AN1792-immunized subjects. **i**, Quantification of average Aβ intensity per cortical region per donor per group. **j**, Representative pTau (AT8) H-DAB staining for each group. **k**, Quantification of cortical AT8 per group. **l**, Pathway enrichment analysis of unique and shared DEGs in Aβ-rich gray matter ST spots (iAD-lim vs. nAD; iAD-ext vs. nAD). **m**, LOESS heatmap showing non-linear gene expression patterns relative to Aβ density in iAD. **n**, LOESS non-linear trajectories relative to Aβ density in iAD. **b-d, i, k**, Bar plots display means ± SEM. **a-d, f-g**, NND = 6; nAD = 6; iAD = 13; iAD-lim = 6, iAD-ext = 7. **i,l**, nAD = 4; iAD = 10; iAD-lim = 6, iAD-ext = 4. **k**, iAD-lim = 5, iAD-ext = 7. l, nAD = 4; iAD-lim = 6; iAD-ext = 4. **m-n**, iAD = 12. **f-g** DESeq2 was used to compare expression levels, with sex, age, average genes detected, and gDNA percentage included as covariates. **b-d, k**, Statistical tests, guided by Shapiro–Wilk and F tests, included t-tests, Mann–Whitney, ANOVA with Tukey’s test, Welch ANOVA with Dunnett’s T3 test, and Kruskal–Wallis with Dunn’s test. **f-g, l**, P-values are FDR-adjusted using the Benjamini-Hochberg correction. *Aβ, amyloid-beta; AN1792-ext, AN1792 immunized with extensive Aβ clearance; AN1792-lim, AN1792 immunized with limited Aβ clearance; DEGs, differentially expressed genes; DESeq2, Differential Expression Analysis for Sequence Count Data (version 2); FCX, frontal cortex; FDR, False Discovery Rate; gDNA, genomic DNA; GM, gray matter; H-DAB, Hematoxylin-3,3’-Diaminobenzidine; iAD, immunized Alzheimer’s disease; LCMB, lecanemab; LOESS, locally estimated scatterplot smoothing; MSigDB, Molecular Signatures Database; MT, mitochondrial; nAD, non-immunized Alzheimer’s disease; nFeatures, number of features; nSpots, number of spatial transcriptomic spots; NND, non-neurologic disease; P-adj, P-value adjusted; PFC, probabilistic fold change; pTau, phosphorylated tau; ST, spatial transcriptomics*.

**Fig. 1:**
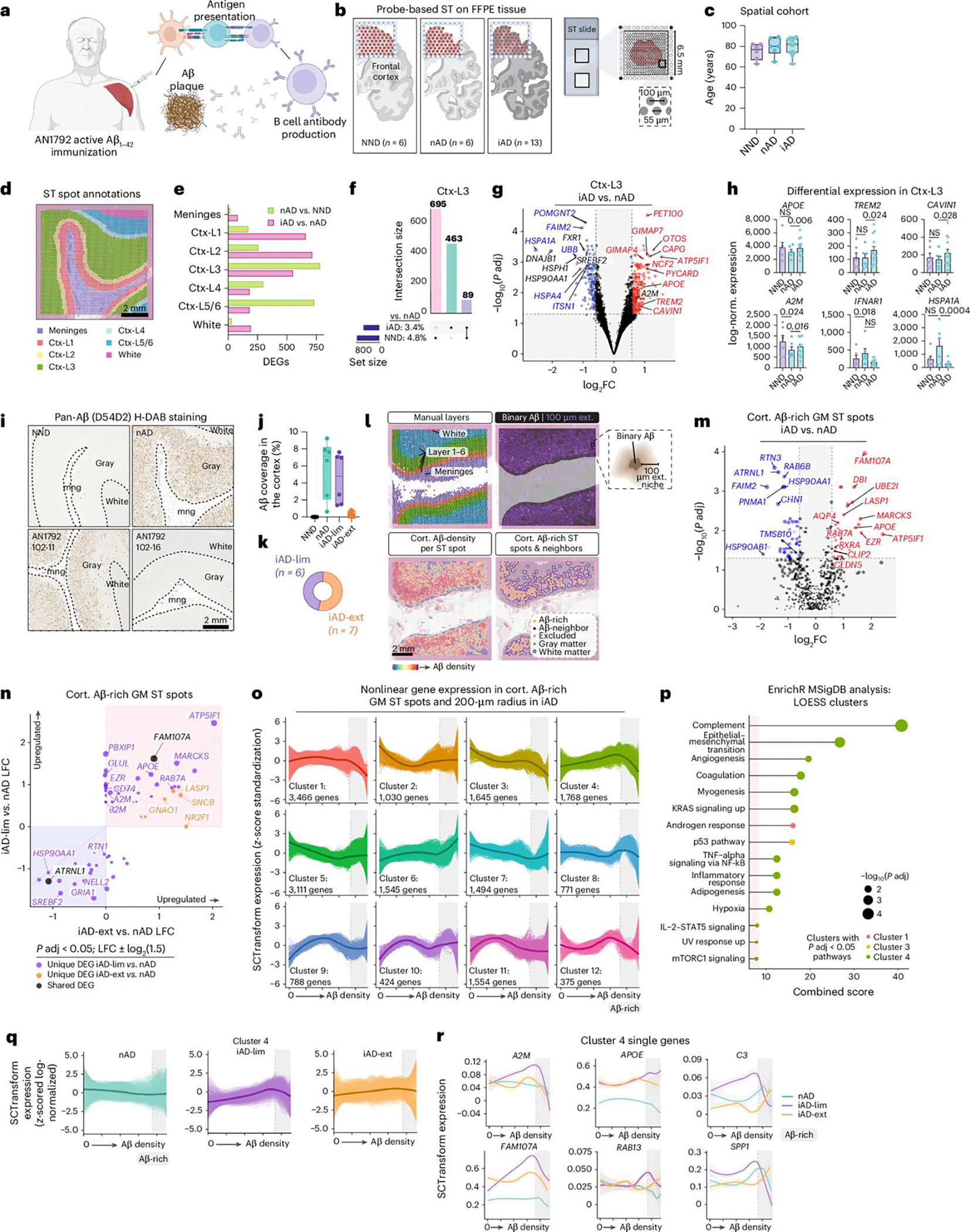
Active Aβ immunization sustains inflammation at the Aβ niche. **a**, AN1792 active Aβ immunization. Created with BioRender.com. **b**, ST method and group sizes of NND, nAD and iAD FCX tissues. Created with BioRender.com. **c**, Study demographics indicating age of each patient. **d**, Manually annotated ST spots in the FCX. **e**, Number of DEGs for each comparison per manually annotated area. **f**, UpSet plot showing unique and shared DEGs across group comparisons in cortical layer III. **g**, DEGs in cortical layer III (iAD versus nAD). Red and blue DEGs are uniquely identified in the iAD versus nAD comparison and are not observed as DEGs in the nAD versus NND comparison. **h**, Pseudobulked expression for various genes in microglia-enriched gray matter ST spots. Error bars indicate the s.e.m. *P* values are from DESeq2. **i**, Representative pan-Aβ H-DAB stains for each group. **j**, Quantification of cortical Aβ coverage per group. **k**, Numbers of iAD-lim and iAD-ext patients among AN1792 actively immunized patients. **l**, Method of processing of Aβ IHC images. The binary Aβ signal was extended by 100 μm beyond its actual size, with a gradual decrease in signal intensity every 20 μm, allowing for detection of genes associated with Aβ density. **m**, DEGs from Aβ-rich gray matter ST spots (iAD versus nAD). **n**, LFC plots for Aβ-rich ST spots in gray matter (iAD-lim versus nAD; iAD-ext versus nAD). **o**, LOESS plots showing clusters of nonlinear gene expression patterns relative to Aβ density in iAD. **p**, Pathway enrichment analysis of genes in nonlinear expression clusters associated with Aβ density in iAD. **q**, LOESS plot of cluster 4 predictions in nAD, iAD-lim and iAD-ext relative to Aβ density. **r**, LOESS plots of select genes in LOESS cluster 4. Dark line indicating the LOESS predicted expression, and light shading represents standard error of the estimated values. **c**,**j**, Box plots are bounded by the 25th and 75th percentiles, the center line shows the median, and whiskers show the data range. **o**,**q**, LOESS plots with the dark line represent the mean LOESS predicted expression per group per cluster, and single lines indicate LOESS predicted gene expression per group per cluster. **c**,**e**–**h**,**j**,**k**, NND = 6; nAD = 6; iAD = 13; iAD-lim = 6, iAD-ext = 7. **m**,**n**, nAD = 4; iAD = 10; iAD-lim = 6, iAD-ext = 4. **o**–**r**, nAD = 4; iAD = 12; iAD-lim = 6, iAD-ext = 6. DESeq2 (**e**–**h**) or MAST (**m** and **n**) was used to compare expression levels. For DESeq2, covariates included sex, age, average genes detected and genomic DNA (gDNA) percentage. In MAST, manually annotated region or cortical layer, sex, age, cellular detection rate (CDR) and gDNA percentage were included as covariates, with sample ID as a random effect. All *P* values were false discovery rate (FDR)-adjusted using Benjamini–Hochberg correction. A2M, alpha-2-macroglobulin; APOE, apolipoprotein E; CAVIN1, caveolae-associated protein 1; Ctx, cortex; FFPE, formalin-fixed paraffin-embedded; GM, gray matter; HSPA1A, heat shock protein family A member 1A; H-DAB, hematoxylin-3,3′-diaminobenzidine; IFNAR1, interferon alpha and beta receptor subunit 1; LFC, log fold change; MSigDB, Molecular Signatures Database; *P* adj, adjusted *P* value. NS, not significant.

We utilized ST to analyze brain frontal cortex (FCX) sections of patients with AD from the AN1792 trial (Fig. 1a). This cohort included 13 iAD brains, as well as 6 nAD and 6 NND control brains (Fig. 1b). Extended Data Table 1 details the clinical and pathologic profiles of these individuals. Groups were age matched (Fig. 1c) and sex matched (Extended Data Fig. 1a). A 6.5 × 6.5-mm ST capture area (4,992 spots) was analyzed, and ST spots were manually annotated by cortical layers, meninges or white matter using H&E staining (Fig. 1d). No significant differences were found in ST spot or feature counts per region (Extended Data Fig. 1b,c). Cortical layer I of iAD samples showed lower mitochondrial gene expression (Extended Data Fig. 1d), suggesting altered mitochondrial metabolism. Annotations were validated by plotting the expression of meningeal, white matter and layer-specific gray matter genes (Extended Data Fig. 1e).

We used a pseudobulk method (DESeq2) to identify differentially expressed genes (DEGs) per region. Deeper cortical layers showed the most DEGs in nAD versus NND controls, while superficial layers were mostly affected in iAD versus nAD controls (Fig. 1e). Cortical layer III exhibited many DEGs in both iAD versus nAD and nAD versus NND comparisons. This high level of transcriptomic dysregulation led us to further examine this cortical layer. Nearly all cortical layer III DEGs were unique to iAD or nAD compared to their controls, with only 7.1% shared (Fig. 1f). These results indicate transcriptomic alterations in superficial layers of the actively immunized AD cortex.

Notably, the triggering receptor expressed on myeloid cells 2 (*TREM2*) and apolipoprotein E (*APOE*) were among the genes upregulated in cortical layer III of the iAD cortex compared to nAD controls (Fig. 1g,h). Both genes are established AD risk factors^19–22^ and are associated with the microglial response to Aβ^23–26^. Additional upregulated cortical layer III genes in iAD versus nAD included alpha-2-macroglobulin (*A2M*) and caveolae-associated protein 1 (*CAVIN1*), involved in inflammation and lysosomal function. Downregulated genes in iAD versus nAD included those encoding heat shock proteins (HSPs), such as heat shock protein family A (*Hsp70*) member 1A (*HSPA1A*) and heat shock protein family H (*Hsp110*) member 1 (*HSPH1*; Fig. 1g,h). HSPs, involved in protein folding^27^ and cellular stress^27,28^, were increased in nAD compared to NND controls (Fig. 1h and Extended Data Fig. 1f). Examination of the most divergent DEGs between iAD versus nAD and nAD versus NND showed that HSP genes were downregulated after immunization but showed the opposite direction in nAD compared to NND (Extended Data Fig. 1g). Conversely, synaptic plasticity-associated genes, such as semaphorin 3G (*SEMA3G*) and Hes family BHLH transcription factor 5 (*HES5*) were upregulated in iAD brains. These findings show transcriptomic alterations in cortical layer III after immunization, including decreased protein folding and stress genes and increased microglial response genes like *APOE* and *TREM2*.

We previously demonstrated Aβ clearance in a subset of AN1792 patients^3–5,16^. To investigate mechanisms driving varying degrees of Aβ clearance in AN1792 patients, we quantified Aβ pathology on sequential slides of ST tissue using immunohistochemistry (IHC; Fig. 1i and Extended Data Fig. 1h). Aβ clearance was most prominent in the superficial cortical layers (Extended Data Fig. 1i), consistent with our previous findings^4^. The iAD cohort was categorized into those with limited (iAD-lim, *N* = 6) and extensive (iAD-ext, *N* = 7) Aβ clearance based on amounts of residual Aβ coverage (Fig. 1j,k). Phosphorylated tau (pTau) was quantified using AT8 load in gray matter across these groups, revealing no significant differences (Extended Data Fig. 1j,k). This dovetails with our prior results showing tau pathology persisted in Aβ-cleared cortical areas^16^.

To capture transcriptomic alterations around Aβ deposits, we overlaid Aβ IHC images from consecutive slides (5–10 μm apart) with ST data and extended the Aβ signal by 100 μm with decreasing intensity every 20 μm (Fig. 1l). This enabled direct examination of the Aβ niche and associated genes. Vascular Aβ-rich ST spots were excluded from analyses. Differential expression analysis of Aβ-rich ST spots in gray matter using model-based analysis of single-cell transcriptomics (MAST)^29^ revealed increased expression of *APOE* and myristoylated alanine-rich C-kinase substrate (*MARCKS*; Fig. 1m) in iAD Aβ niches. *MARCKS* is expressed in activated microglia that surround Aβ plaques^30^. The most upregulated gene at the Aβ niche was family with sequence similarity 107 member A (*FAM107A*), a stress-responsive actin-bundling factor influencing synaptic efficiency and cognition^31^.

Comparison of the Aβ niche between iAD-lim and iAD-ext brains revealed upregulation of inflammatory genes like beta-2-microglobulin (*B2M*), *A2M*, *CD74* molecule (*CD74*), *APOE* and *MARCKS* in iAD-lim but not iAD-ext brains (Fig. 1n). Pathway analyses revealed enrichment of interferon alpha response and interleukin-2 (IL-2)–STAT5 signaling in the iAD-lim Aβ niche (Extended Data Fig. 1l). Collectively, genes altered in Aβ-rich ST spots show an inflammatory signature within the Aβ niche of iAD-lim versus iAD-ext.

We visualized nonlinear relationships between gene expression and Aβ density in Aβ-rich ST spots (200-μm radius) using locally estimated scatterplot smoothing (LOESS; Extended Data Fig. 1m,n). Hierarchical clustering delineated distinct expression patterns with increasing Aβ density within the iAD group (Fig. 1o). Pathway analysis of gene clusters revealed that cluster 4, peaking in Aβ-rich ST spots, was enriched for immune-related pathways, including complement signaling, inflammatory response and IL-2–STAT5 signaling (Fig. 1p). Cluster 4 genes showed the highest upregulation in iAD-lim Aβ niches, a lesser increase in iAD-ext and no upregulation in nAD (Fig. 1q). This cluster contained many immune-associated genes, including *A2M*, *APOE*, complement C3 (*C3*), member RAS oncogene family (*RAB13*) and secreted phosphoprotein 1 (*SPP1*; Fig. 1r). Together, these findings reveal sustained inflammation at the Aβ niche in AN1792-immunized brains with limited Aβ clearance, marked by IL-2–STAT5 and complement signaling, and upregulation of inflammatory response genes in AD.

### Microglial phenotypes define varying degrees of Aβ clearance

**Extended Data Fig. 2:**
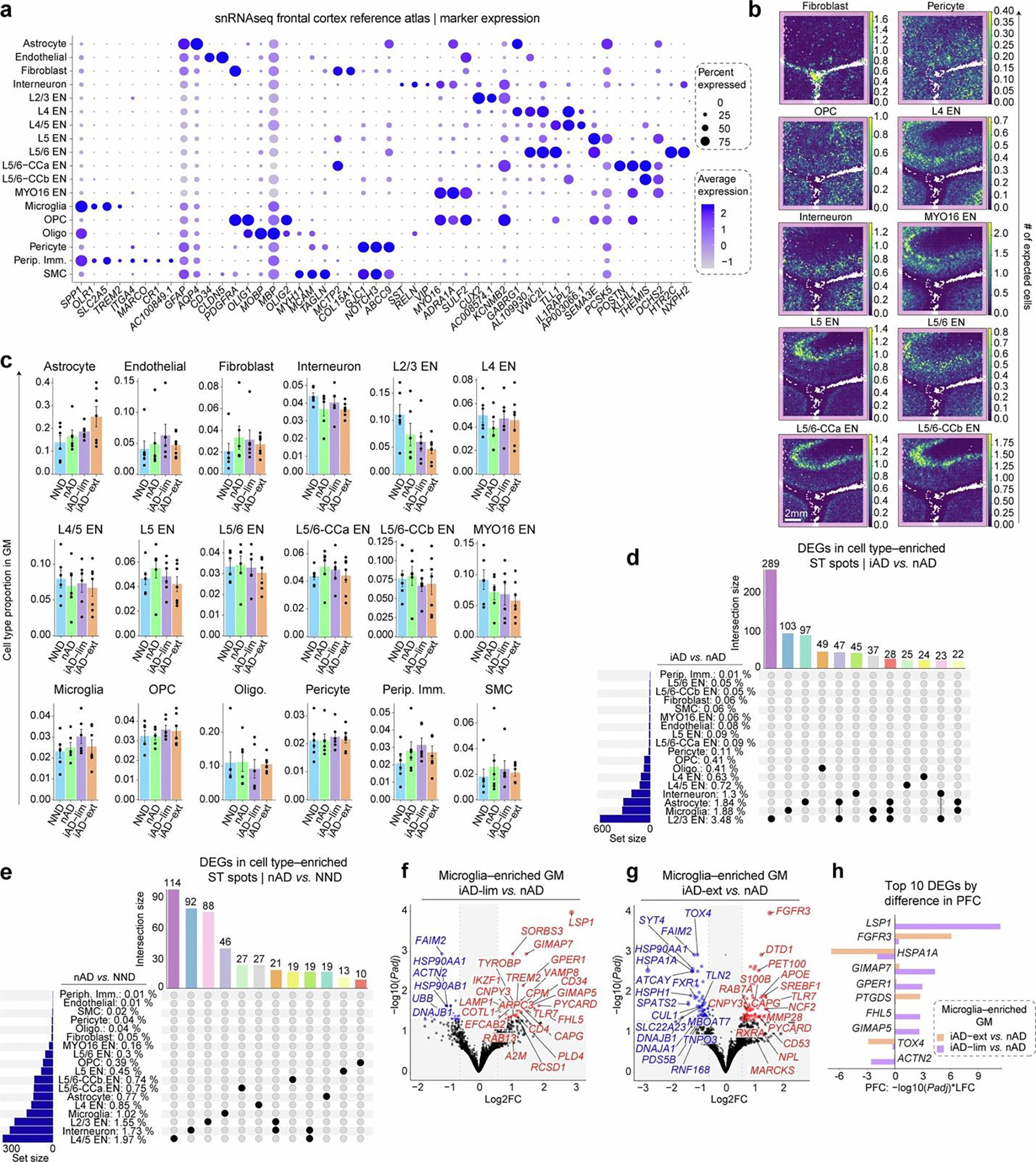
Microglial phenotypes define varying degrees of Aβ clearance. **a**, Bubble plot heatmap showing top markers expressed by cell types in the reference atlas. **b**, Spatial plots displaying the abundance of deconvoluted cell types. **c**, Bar graphs showing the proportion of deconvoluted cell types in gray matter per donor, per group. Statistical tests, guided by Shapiro–Wilk and F tests, included ANOVA with Tukey’s test, Welch ANOVA with Dunnett’s T3 test, and Kruskal–Wallis with Dunn’s test**. d**, UpSet plot indicating unique and shared DEGs in deconvoluted cell types for iAD vs. nAD. **e**, UpSet plot showing unique and shared DEGs in deconvoluted cell types for nAD vs. NND. **f-g**, Volcano plot of DEGs in microglia-enriched spots in: **f**, iAD-lim vs. nAD; and **g**, iAD-ext vs. nAD. **h**, Bar plot of the top 10 most divergent DEGs in microglia-enriched ST spots based on PFC, comparing iAD-lim vs. nAD and iAD-ext vs. nAD. Adjusted P-values used to calculate PFC are derived from DESeq2. **c**, Bar plots display means ± SEM**. a**, 424 ROSMAP DLPFC samples. **c-h**, NND = 6; nAD = 6; iAD = 13; iAD-lim = 6, iAD-ext = 7**. d-h**, DESeq2 was used to compare expression levels, with sex, age, average genes detected, and gDNA percentage included as covariates. All P-values are FDR-adjusted using the Benjamini-Hochberg correction. *Aβ, amyloid-beta; AD, Alzheimer’s disease; AN1792-ext, AN1792 immunized with extensive Aβ clearance; AN1792-lim, AN1792 immunized with limited Aβ clearance; DEGs, differentially expressed genes; DESeq2, Differential Expression Analysis for Sequence Count Data (version 2); DLPFC, dorsolateral prefrontal cortex; EN, excitatory neuron; FDR, False Discovery Rate; gDNA, genomic DNA; GM, gray matter; iAD, immunized Alzheimer’s disease; L, layer; LCMB, lecanemab; nAD, non-immunized Alzheimer’s disease; NND, non-neurologic disease; oliog., oligodendrocyte; OPC, oligodendrocyte precursor cell; P-adj, P-value adjusted; periph. imm., peripheral immune cell; PFC, probabilistic fold change; ROSMAP, Religious Orders Study and Rush Memory and Aging Project; SEM, standard error of the mean; SMC, smooth muscle cell; snRNA-seq, single-nucleus RNA sequencing; ST, spatial transcriptomics*.

**Fig. 2:**
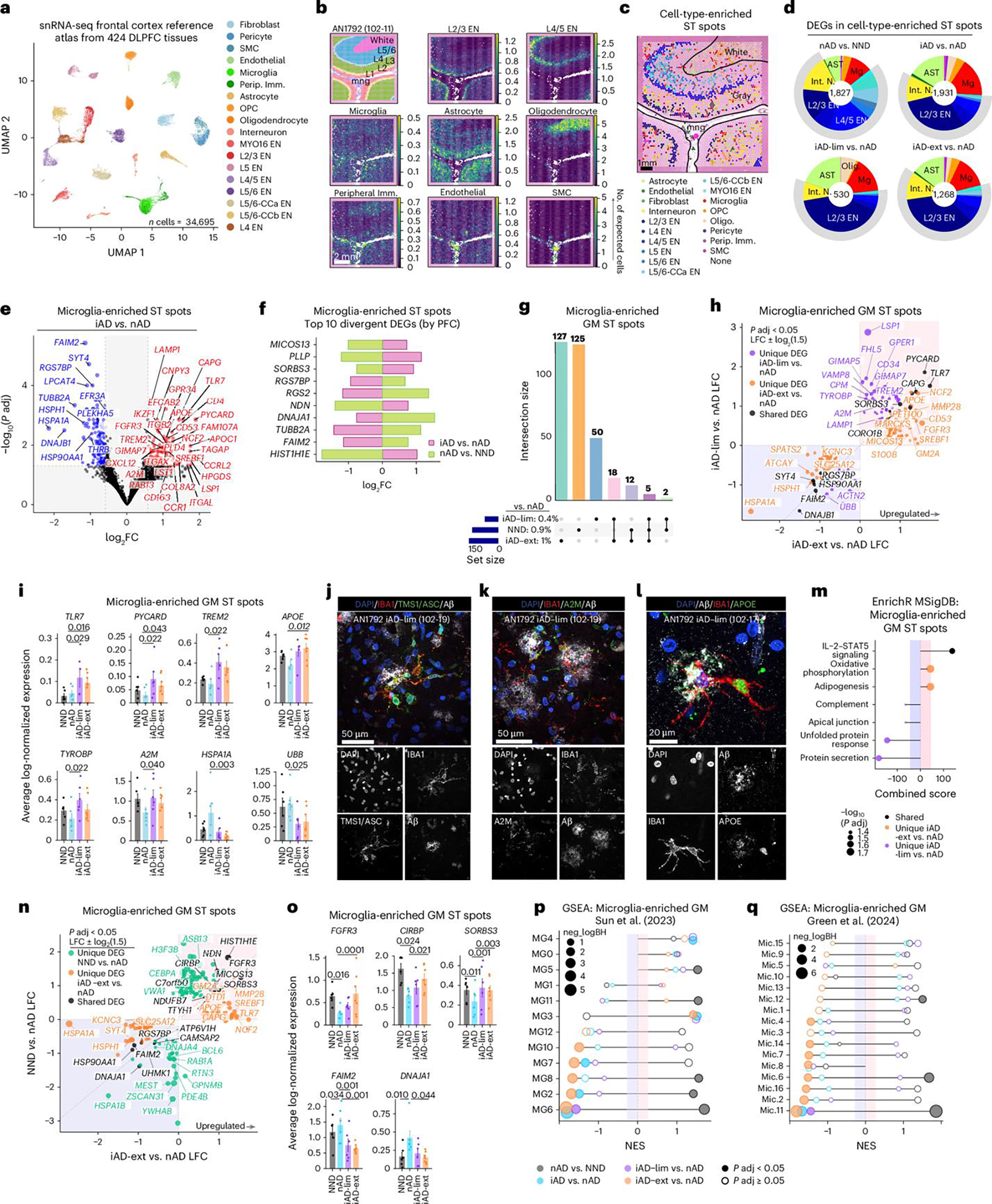
Microglial phenotypes define varying degrees of Aβ clearance. **a**, Reference atlas UMAP from DLPFC snRNA-seq data^33,34^. **b**, Spatial plots showing abundance of deconvoluted cell types. **c**, Spatial plots highlighting enriched ST spots for deconvoluted cell types. **d**, Percentages of DEGs expressed in enriched ST spots per cell type: nAD versus NND, iAD versus nAD, iAD-lim versus nAD and iAD-ext versus nAD. The number in the center of each pie chart represents the total number of DEGs. **e**, DEGs from microglia-enriched ST spots (iAD versus nAD). **f**, Top ten divergent DEGs in microglia-enriched ST spots based on PFC, comparing iAD versus nAD and nAD versus NND. **g**, UpSet plot showing unique and shared DEGs in microglia-enriched ST spots in gray matter across groups compared to nAD. **h**, LFC plots for microglia-enriched ST spots in gray matter (iAD-lim versus nAD; iAD-ext versus nAD). **i**, Pseudobulked expression for various genes in microglia-enriched ST gray matter spots. Error bars show the s.e.m. *P* values are from DESeq2. **j**–**l**, Confocal images showing TMS1/ASC^+^IBA1^+^ myeloid cells (**j**), A2M^+^IBA1^+^ myeloid cells (**k**) and APOE^+^IBA1^+^ myeloid cells (**l**) around Aβ deposits in the FCX of iAD. **m**, Pathway enrichment analysis of unique and shared DEGs in microglia-enriched gray matter ST spots (iAD-lim versus nAD; iAD-ext versus nAD). **n**, LFC plots for microglia-enriched gray matter ST spots (NND versus nAD; iAD-ext versus nAD). **o**, Pseudobulked expression for various genes in microglia-enriched ST spots in gray matter. Error bars show the s.e.m. *P* values are from DESeq2. **p**–**q**, Pathway enrichment analysis of predefined microglial states from **p**^41^ and **q**^34^, using genes ranked by PFC in iAD-lim versus nAD and iAD-ext versus nAD. **i**,**o**, Bar plots display means ± s.e.m. **d**–**i**,**m**–**q**, NND = 6; nAD = 6; iAD = 13; iAD-lim = 6, iAD-ext = 7. DESeq2 was used to compare expression levels, with sex, age, average genes detected and gDNA percentage included as covariates (**d**–**i** and **n**–**o**). All *P* values were FDR adjusted using Benjamini–Hochberg. Ast, astrocyte; CCa, cortico-cortical cluster a; CCb, cortico-cortical cluster b; CIRBP, cold-inducible RNA-binding protein; FAIM2, Fas apoptotic inhibitory molecule 2; FGFR3, fibroblast growth factor receptor 3; GSEA, gene-set enrichment analysis; IBA1, ionized calcium-binding adapter molecule 1; Int. N., interneuron; L, layer; Mg, microglia; NES, normalized enrichment score; OPC, oligodendrocyte precursor cell; Perip. Imm., peripheral immune cells; PYCARD, PYD and CARD domain containing; SMC, smooth muscle cell; SORBS3, sorbin and SH3 domain containing 3; TLR7, Toll-like receptor 7; TYROBP, TYRO protein tyrosine kinase-binding protein; UBB, ubiquitin B; UMAP, uniform manifold approximation and projection.

Because ST spots encompass 1–10 cells each, we aimed to resolve their cellular composition using Cell2Location (C2L)^32^, which integrates ST data with single-nucleus RNA sequencing (snRNA-seq). We constructed a reference atlas from a snRNA-seq dataset of 424 dorsolateral prefrontal cortex (DLPFC) tissues from AD and NND controls^33,34^, downsampling to 34,695 cells for near-equal cell-type representation (Fig. 2a and Extended Data Fig. 2a). C2L analysis mapped cell types to expected spatial locations (Fig. 2b and Extended Data Fig. 2b). In the gray matter after AN1792 immunization, excluding layer I due to unreliable cell mapping, we observed increased relative numbers of astrocytes and reduced layer 2/3 (L2/3) excitatory neurons (ENs), although these changes were not statistically significant. Microglia were predicted to be most abundant in the iAD-lim cortex (Extended Data Fig. 2c).

We then annotated the most highly enriched ST spots for specific cell types within their expected spatial regions (Fig. 2c). Comparing iAD to nAD gene expression in cell-type-enriched ST spots showed most DEGs in L2/3 EN-enriched ST spots, followed by microglia and astrocytes (Fig. 2d and Extended Data Fig. 2d). Conversely, nAD samples showed DEGs predominantly in layer 4/5 (L4/5) ENs, interneurons and L2/3 ENs when compared to NND (Fig. 2d and Extended Data Fig. 2e). Microglia-enriched ST spots in iAD versus nAD showed the upregulation of *APOE*, *TREM2*, *A2M*, *RAB13*, *FAM107A* and other amyloid-response genes such as lysosomal-associated membrane protein 1 (*LAMP1*), CD163 molecule (*CD163*), PYD and CARD domain containing (*PYCARD*), integrin subunit alpha X (*ITGAX*) and apolipoprotein C1 (*APOC1*), while HSP genes were downregulated (Fig. 2e). Top divergent DEGs between iAD versus nAD and nAD versus NND (for example, DnaJ heat shock protein family *(Hsp40)* member A1 (*DNAJA1*) and Fas apoptotic inhibitory molecule 2 (*FAIM2*)) indicated reduced cellular stress and disrupted apoptosis after AN1792 (Fig. 2f).

Differential expression analysis of microglia-enriched ST spots revealed more DEGs in iAD-ext than in iAD-lim compared to nAD, indicating a more distinct microglial state with extensive Aβ clearance (Fig. 2g and Extended Data Fig. 2f,g). Unique iAD-ext upregulated genes included *APOE*, *MARCKS* and fibroblast growth factor receptor 3 (*FGFR3*; Fig. 2h). FGFR3 serves as a receptor for fibroblast growth factor 2 (FGF2), which neurons release in response to oligomeric Aβ-induced damage. This interaction promotes microglial migration and debris phagocytosis, aiding in neuroprotection^35^. In iAD-lim, we found upregulation of previously mentioned *TREM2*, *A2M* and *LAMP1*, as well as transmembrane immune signaling adaptor TYROBP (*TYROBP*), which links TREM2 to *APOE* transcription in microglia^36^. Shared upregulated genes included *PYCARD* and Toll-like receptor 7 (*TLR7*; Fig. 2h and Extended Data Fig. 2f,g), while non-shared genes showed similar trends without significance in both groups (Fig. 2i). PYCARD activates the inflammasome, forming apoptosis-associated speck-like protein containing a CARD (ASC) specks that can cross-seed Aβ pathology^37^. Localization of proteins TMS1/ASC (encoded by *PYCARD*), A2M and APOE was confirmed in IBA1^+^ microglia around Aβ plaques in the AN1792-immunized FCX (Fig. 2j–l).

Most divergent genes in microglia-enriched ST spots between Aβ clearance groups were leukocyte-specific protein 1 (*LSP1*) and GTPase of immunity-associated protein (*GIMAP*) genes in iAD-lim and *FGFR3* and *HSPA1A* in iAD-ext (Extended Data Fig. 2h). LSP1 localizes to nascent phagocytic cups during Fcγ-receptor-mediated phagocytosis^38^. Pathway analysis revealed increased IL-2–STAT5 signaling in both groups, with iAD-ext uniquely upregulating oxidative phosphorylation and adipogenesis, while iAD-lim showed downregulation of complement and unfolded protein response pathways (Fig. 2m).

We next examined genes shared between iAD-ext versus nAD and NND versus nAD in microglia-enriched regions to determine if transcriptomic changes in iAD-ext brains reflect a return to homeostasis similar to NND. Shared upregulated genes included *FGFR3*, cold-inducible RNA-binding protein (*CIRBP*) and sorbin and SH3 domain containing 3 (*SORBS3*), while downregulated genes included *FAIM2*, *DNAJA1* and heat shock protein 90 alpha family class A member 1 (*HSP90AA1*; Fig. 2n,o). *CIRBP* is a stress-responsive gene that modulates inflammation^39^ and ameliorates neuronal amyloid toxicity via antioxidative and antiapoptotic pathways^40^. These findings suggest that some DEGs in microglia-enriched ST spots of iAD-ext brains reflect a shift in microglial gene expression toward an NND control profile.

To investigate microglial function after AN1792 immunization, we compared microglia-enriched ST spot signatures of iAD brains to published human AD microglial states34^,^41. Here, iAD brains after AN1792 immunization showed reduced stress-responsive microglia (MG6), inflammatory states (MG2, MG8, MG10) and glycolytic microglia (MG7), and increased ribosome biogenesis microglia (MG3; Fig. 2p). Notably, MG3 microglia exhibit strong enrichment of disease-associated microglia (DAM) signature genes^41^. In contrast, inflammatory and stress-responsive microglia states were increased in nAD versus NND. Using separate microglial classifications^34^ showed a reduction in stress-responsive microglia (Mic.11), surveilling microglia (Mic.2, Mic.4), reactive microglia (Mic.6–Mic.8), interferon-responsive microglia (Mic.14) and serpin family E member 1 (*SERPINE1*)-expressing microglia (Mic.16) after immunization (Fig. 2q).

Overall, active Aβ immunization reduces stress-responsive microglia irrespective of residual Aβ levels. Yet, microglia in iAD-ext shifted from glycolysis to oxidative phosphorylation, while iAD-lim showed decreased complement and unfolded protein responses with upregulated phagocytosis genes. These findings suggest that effective Aβ clearance relies on balanced microglial metabolic states that also protect against Aβ neurotoxicity.

### Passive Aβ immunization induces distinct microglial states

**Fig. 3:**
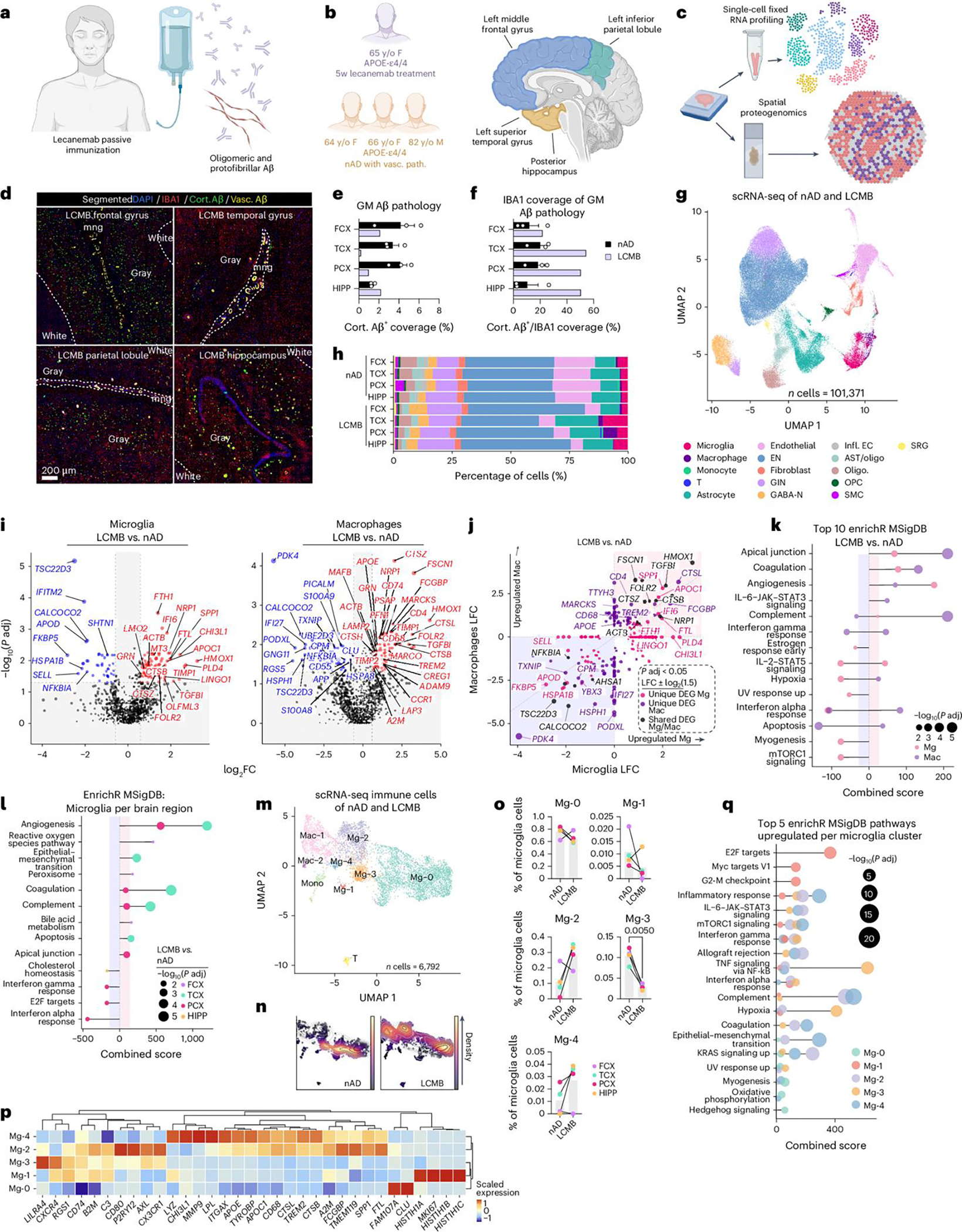
Passive Aβ immunization induces distinct microglial states. **a**, Lecanemab binds oligomeric and protofibrillar Aβ to promote Aβ clearance from the brain. Created with BioRender.com. **b**, Study participants included a 65-year-old female patient with AD who was treated with lecanemab and three matched nAD controls. Tissues analyzed included cortical areas and HIPP. Created with BioRender.com. **c**, Tissues were analyzed by scRNA-seq and spatial proteogenomics. Created with BioRender.com. **d**, Confocal images showing segmented Aβ burden and microgliosis in regions of the lecanemab-treated patient brain. **e**, Percentage of cortical Aβ coverage in brain regions from the lecanemab case and nAD controls. **f**, Percentage of cortical Aβ covered by IBA1. **g**, UMAP showing annotated cell types. **h**, Percentages of each cell type for each brain region between nAD controls and lecanemab case. **i**, DEGs in microglia and macrophages comparing lecanemab to nAD. **j**, LFC plots comparing DEGs in microglia and macrophages (lecanemab versus nAD). **k**, Top ten pathway enrichment analysis of DEGs in microglia and macrophages (lecanemab versus nAD). **l**, Pathway enrichment analysis of DEGs in microglia from FCX, TCX, PCX and HIPP (lecanemab versus nAD). **m**, Clustering of microglia from scRNA-seq of the lecanemab case and nAD controls. **n**, UMAP density plots showing microglial cluster distribution for the lecanemab case and nAD controls. **o**, Percentages of microglial clusters in the lecanemab case versus nAD controls. Normality tests dictated if *P* values were calculated using a two-tailed paired *t*-test or Wilcoxon test. **p**, Marker genes for each microglial cluster. **q**, Top five upregulated pathways using marker genes defining the microglial states. **e**,**f**, Bar plots display means ± s.e.m. **o**, Bar plots display means. **e**,**f**,**o**, Statistical tests, guided by Shapiro–Wilk and *F* tests, included *t*-tests, Mann–Whitney tests (**e** and **f**) and paired *t*-tests (**o**). **e**–**q**, nAD = 3; LCMB = 1. **i**,**j**, MAST was used to compare expression levels, with brain region and CDR as covariates and brain region and sample ID included as a random effect. **i**–**l**,**q**, *P* values were FDR adjusted using Benjamini–Hochberg. Cort., cortical; GABA-N, GABAergic neuron; GIN, GABAergic interneuron; Infl. ECs, inflamed endothelial cells; LCMB, lecanemab; Mac, macrophages; mng, meninges; Oligo, oligodendrocyte; SRG, stress-responsive glia; Vasc, vascular.

Intrigued by the microglial response to Aβ in actively immunized AD brains, we extended our investigation to examine immune responses to passive lecanemab immunization (Fig. 3a). We analyzed a unique case of a patient with AD who received three lecanemab infusions over 5 weeks, shortly before passing away from intracerebral hemorrhages^17,18^. Our postmortem analysis revealed histiocytic vasculitis in CAA-affected vessels, with vascular Aβ fragmentation and phagocytosis across the cortex, alongside a ‘high’ burden of AD pathology per National Institute on Aging and Alzheimer’s Association (NIA-AA) guidelines^17,18^. Notably, parenchymal Aβ plaque phagocytosis was also observed^18^. We compared this patient to three *APOE* ε4/ε4-matched controls with high AD pathology and vascular AD pathology without anti-Aβ treatment (Fig. 3b and Extended Data Table 2). Cortical tissues studied were from the left middle FCX, superior temporal cortex (TCX) and inferior parietal lobule (PCX), as well as the posterior hippocampus (HIPP). These regions were selected for their varying levels of Aβ clearance in this patient brain^18^. We used scRNA-seq and spatial proteogenomics to identify cell-type-specific immune responses to passive Aβ immunization in these regions (Fig. 3c).

**Extended Data Fig. 3:**
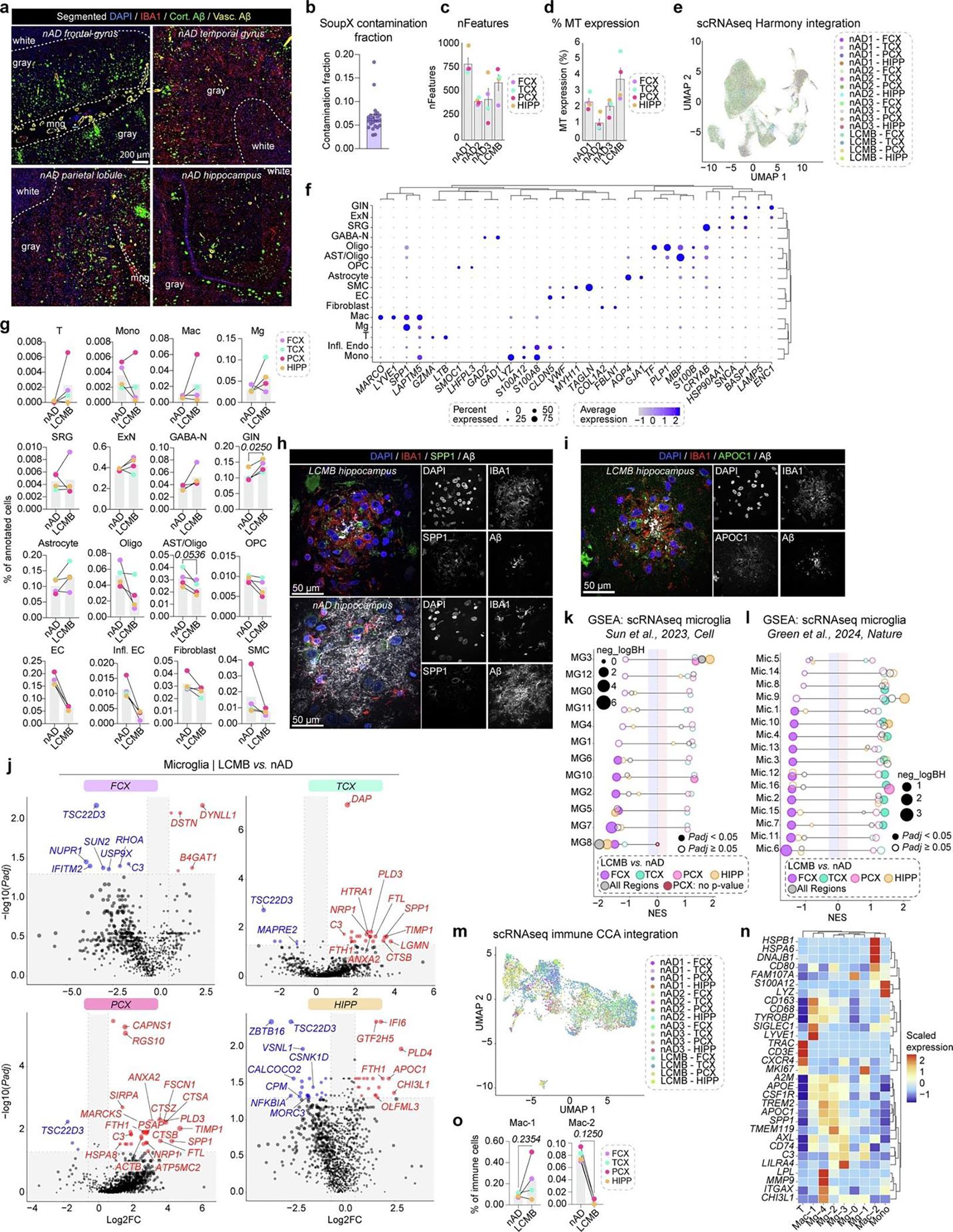
Passive Aβ immunization induces distinct microglial states. **a**, Representative confocal images showing segmented Aβ burden and microgliosis in regions of the nAD control patient’s brain. **b**, SoupX contamination fraction for each scRNA-seq sample. **c**, Average number of features (genes) per cell per donor. **d**, Percentages of mitochondrial gene per cell averaged per donor**. e**, Integrated scRNA-seq dataset showing all analyzed cells from nAD controls and lecanemab case. **f**, Bubble plot heatmap of top markers expressed by cell types in the scRNA-seq dataset. **g**, Changes in percentages of total annotated cells for each cell type**. h**, Confocal images showing SPP1^+^IBA1^+^ myeloid cells surrounding Aβ deposits in the hippocampus of the lecanemab-treated patient, absent in the nAD control. **i**, Confocal images showing APOC1^+^IBA1^+^ myeloid cells surrounding Aβ deposits in the hippocampus of the lecanemab-treated patient. **j**, Volcano plot of DEGs from scRNA-seq microglia (lecanemab vs. nAD) in FCX (top left), TCX (top right), PCX (bottom left), and HIPP (bottom right). **k-l**, Pathway enrichment analysis of predefined microglial states from **k**, Sun et al.^41^, and **l, G**reen et al.^34^, using genes ranked by PFC in scRNA-seq regional microglia (lecanemab vs. nAD). **m**, UMAP showing reintegrated scRNA-seq immune cells for each brain region in the lecanemab case and nAD controls**. n**, Marker genes for each immune cell cluster. **o**, Percentages of each macrophage cluster. **b-d**, Bar plots display means ± SEM. **g, o**, Bar plots display means. b-e, g, j-o, nAD = 3; LCMB = 1. **j**, MAST was used to compare expression levels, with CDR as a covariate and brain region * sample ID included as a random effect. **c-d, g, o**, Statistical tests, guided by Shapiro–Wilk and F tests, included t-tests, Mann–Whitney, ANOVA with Tukey’s test, Welch ANOVA with Dunnett’s T3 test, and Kruskal–Wallis with Dunn’s test. **j-l**, P-values are FDR-adjusted using the Benjamini-Hochberg correction. *Aβ, amyloid-beta; AD, Alzheimer’s disease; APOC1, apolipoprotein C1; Ast, astrocyte; CCA, canonical correlation analysis; CDR, Cellular Detection Rate; DEGs, differentially expressed genes; EC, endothelial cells; ExN, excitatory neuron; FCX, frontal cortex; FDR, False Discovery Rate; GABA-N, GABAergic neuron; gDNA, genomic DNA; GIN, GABAergic interneuron; HIPP, hippocampus; IBA1, ionized calcium-binding adapter molecule 1; Infl. EC, inflamed endothelial cells; LCMB, lecanemab; Mac, macrophages; MAST, Model-based Analysis of Single-cell Transcriptomics; Mg, microglia; Mono, monocytes; MT, mitochondrial; nAD, non-immunized Alzheimer’s disease; nFeatures, number of features; Oligo, oligodendrocyte; OPC, oligodendrocyte precursor cell; P-adj, P-value adjusted; PCX, parietal cortex; PFC, probabilistic fold change; scRNA-seq, single-cell fixed RNA sequencing; SEM, standard error of the mean; SMC, smooth muscle cell; SPP1, secreted phosphoprotein 1; SRG, stress-responsive glia; ST, spatial transcriptomics; TCX, temporal cortex; UMAP, uniform manifold approximation and projection; Vasc., vascular*.

Tissue sections were stained for IBA1 and pan-Aβ. Using manual annotation and machine learning, we differentiated cortical and vascular Aβ pathology (Fig. 3d and Extended Data Fig. 3a). Gray matter Aβ quantification showed reduced cortical Aβ in the TCX and PCX of the lecanemab case compared to controls (Fig. 3e). Moreover, a higher fraction of cortical Aβ (~44%) was covered by IBA1^+^ cells in the lecanemab case versus ~15% in controls (Fig. 3f). These data indicate regional variability in Aβ clearance by myeloid cells.

To further explore immune responses to Aβ following passive immunization, we performed scRNA-seq on cells isolated from each brain region, and used SoupX^42^ to minimize ambient RNA contamination (Extended Data Fig. 3b). We assessed quality-control metrics (Extended Data Fig. 3c,d), integrated cells from all tissues (Extended Data Fig. 3e) and annotated cell clusters using their highly expressed genes (Fig. 3g and Extended Data Fig. 3f). The lecanemab case showed a relative increase in GABAergic interneurons and a decrease in endothelial cells, fibroblasts and smooth muscle cells (Fig. 3h and Extended Data Fig. 3g). T cells were enriched in all regions except HIPP, monocytes/macrophages in PCX and TCX, and microglia in TCX, PCX and HIPP (Fig. 3h and Extended Data Fig. 3g).

Differential expression analysis of microglia and macrophages revealed upregulated genes linked to microglial activation (*SPP1* and chitinase 3 like 1 (*CHI3L1*)), lysosomal function (cathepsin B (*CTSB*), granulin (*GRN*)), and interferon response (interferon alpha inducible protein 6 (*IFI6*) in the lecanemab case (Fig. 3i). Additional upregulated genes included those linked to iron storage (ferritin heavy chain 1 (*FTH1*), ferritin light chain (*FTL*)) and lipid metabolism (*APOC1*; Fig. 3i). *SPP1* and *APOC1* were the most upregulated genes unique to microglia (Fig. 3j). *SPP1* is expressed by activated-response microglia^43^ and contributes to tissue repair^44^. We confirmed expression of SPP1 and APOC1 proteins in plaque-associated microglia within the HIPP following lecanemab treatment (Extended Data Fig. 3h,i). Macrophage-specific upregulated genes included *TREM2*, *APOE* and the phagocytosis-associated gene cluster of differentiation 68 (CD68) molecule (*CD68*; Fig. 3j). Both cell types exhibited decreased HSP gene expression, while heme oxygenase 1 (*HMOX1*), the most upregulated gene shared between them, reflected an immune response to hemorrhages in the lecanemab case.

To study microglia and macrophage functions after immunization, we performed enrichment analysis. Pathways regulating vascular functions such as apical junctions, coagulation and angiogenesis were upregulated in macrophages and microglia in the lecanemab case (Fig. 3k). Additionally, we found dysregulated complement signaling in macrophages and increased complement signaling in microglia (Fig. 3k). We also observed dysregulated IL-2–STAT5 signaling in microglia, with both downregulated and upregulated DEGs associated with this pathway (Fig. 3k). These data highlight distinct alterations to the brain myeloid compartment following passive Aβ immunization.

Notably, microglial transcriptomic signatures in the lecanemab case varied by brain region (Extended Data Fig. 3j). Most changes in microglial gene expression were observed in the TCX and PCX, the two regions with the most Aβ clearance. Microglia from these regions exhibited increased expression of genes involved in complement signaling (*C3*), lysosomal function and protein degradation (for example, cathepsin genes), iron storage and regulation (*FTH1*, *FTL*) and *SPP1* (Extended Data Fig. 3j). Regional DEGs were associated with various signaling pathways. In the FCX, DEGs indicated increased reactive oxygen species signaling (Fig. 3l). The TCX and PCX showed increased complement signaling, while the PCX also exhibited decreased interferon responses, among other changes (Fig. 3l). The HIPP demonstrated decreased cholesterol homeostasis (Fig. 3l). Additionally, microglial DEGs were linked to vascular pathways (for example, angiogenesis and coagulation), but this association was present only in the TCX and PCX, areas with extensive Aβ clearance (Fig. 3l). Thus, distinct microglial phenotypes may underlie the variability in Aβ clearance between brain regions of the lecanemab case.

Microglial states^41^ were also altered, with reduced inflammatory MG8 microglia and increased ribosomal biogenesis MG3 microglia across brain regions in the lecanemab case (Extended Data Fig. 3k). In the FCX, where IBA1-Aβ recruitment was low, reductions were seen in inflammatory (MG2, MG8, MG10), phagocytic (MG5), stress-signature (MG6) and glycolytic (MG7) microglia. Conversely, inflammatory MG10 microglia increased in the PCX where IBA1-Aβ recruitment and Aβ clearance were high. Separate microglial classifications^34^ showed a similar pattern with several microglial states downregulated in the FCX, that were upregulated in the TCX, PCX and HIPP regions (Extended Data Fig. 3l). These findings reveal a reduction in inflammatory MG8 microglia and an increase in ribosome biogenesis and DAM-expressing MG3 microglia across all brain regions after lecanemab immunization, similar to AN1792. Notably, the FCX displayed a distinct microglial profile compared to other brain regions.

Immune cell sub-clustering identified two microglial states, Mg-2 and Mg-4, that were enriched in lecanemab-treated brain regions with most IBA1-Aβ recruitment and Aβ clearance (Fig. 3m–o and Extended Data Fig. 3m–o). Mg-2 exhibited a mixed DAM and homeostatic profile, expressing *TREM2*, *APOE* and homeostatic markers, along with high levels of AXL receptor tyrosine kinase (*AXL*), *C3, CD74* and *SPP1* (Fig. 3o,p). Mg-4 displayed a classic DAM signature, with elevated *ITGAX*, lipoprotein lipase *(LPL)*, matrix metallopeptidase 9 (*MMP9*), *CHI3L1* and *SPP1* (Fig. 3o,p). Both clusters showed enhanced complement pathway signaling (Fig. 3q).

In summary, we identified upregulated genes (for example, *SPP1* and *APOC1*) in microglia after lecanemab treatment. Additionally, we observed two distinct microglial phenotypes in brain regions with Aβ clearance, both expressing *APOE* and *TREM2*, and showing increased complement signaling. These findings demonstrate that passive Aβ immunization triggers specific microglial adaptations associated with Aβ clearance.

### Spatial proteogenomics links the Aβ niche to microglial states

**Extended Data Fig. 4:**
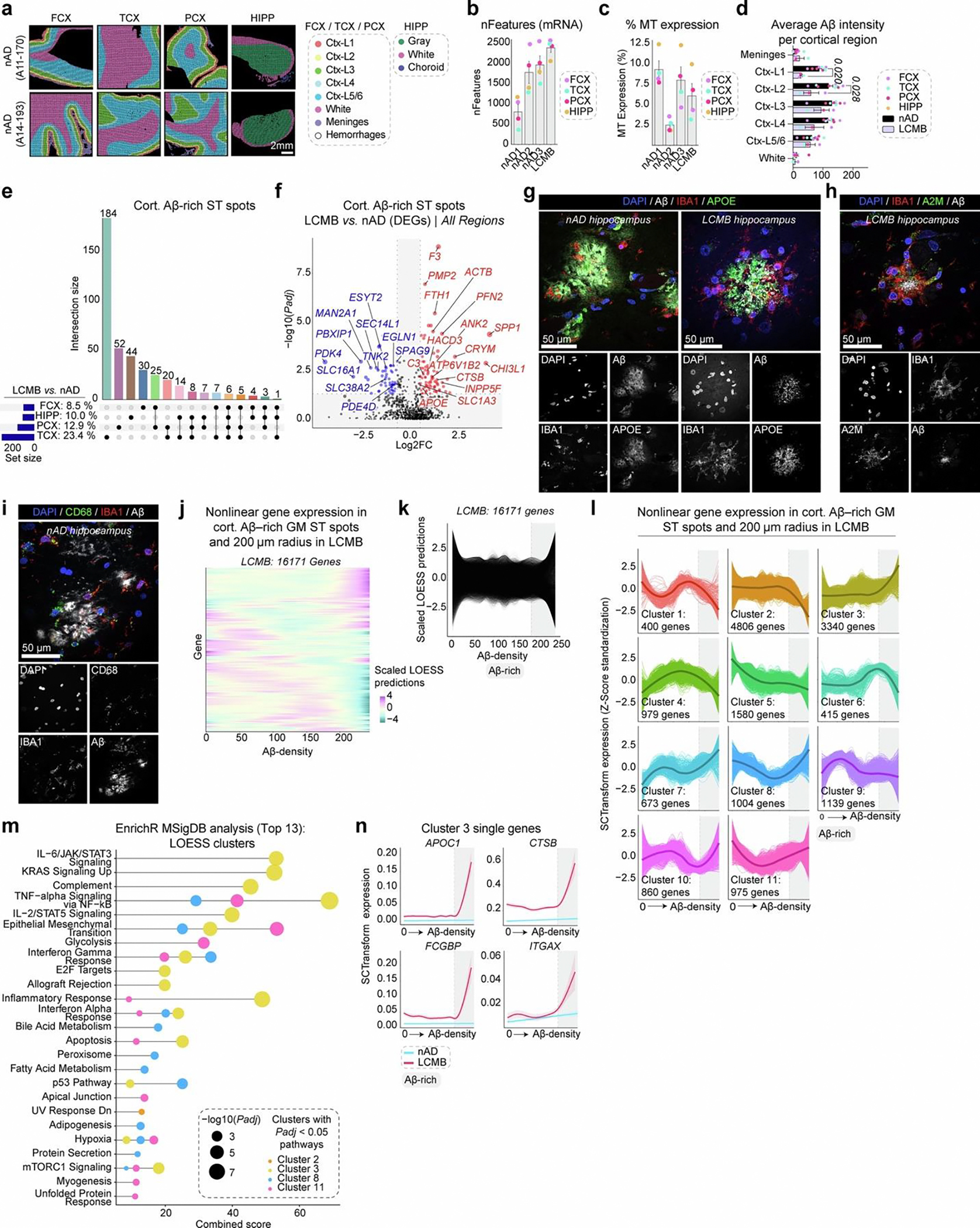
Spatial proteogenomics links the Aβ niche to microglial states. **a**, Manual annotations of analyzed brain regions. **b**, Average number of features (genes) per spot per manually annotated area per donor. **c**, Percentages of mitochondrial gene expression per spot averaged per manually annotated area per donor. **d**, Quantification of average Aβ intensity per cortical region per donor per group. **e**, UpSet plot indicating unique and shared DEGs in cortical Aβ-rich ST spots in FCX, TCX, PCX and HIPP for lecanemab vs. nAD. **f**, Volcano plot of DEGs from Aβ-rich gray matter ST spots (lecanemab vs. nAD) across all regions. **g**, Confocal images showing IBA1^+^ myeloid cells surrounding Aβ deposits that colocalize with APOE in the hippocampus of the lecanemab-treated patient, with reduced IBA1^+^ recruitment in the nAD control. **h**, Confocal images showing A2M^+^IBA1^+^ myeloid cells surrounding Aβ deposits in the hippocampus of the lecanemab-treated patient. **i**, Confocal images showing CD68+IBA1+ myeloid cells surrounding Aβ deposits in the hippocampus of the nAD control. **j**, LOESS heatmap showing non-linear gene expression patterns relative to Aβ density in lecanemab**. k**, LOESS non-linear trajectories relative to Aβ density in lecanemab. **l**, LOESS plots showing clusters of non-linear gene expression patterns relative to Aβ density in lecanemab. Dark line representing the mean LOESS predicted expression for the cluster and single lines indicating LOESS predicted gene expression per cluster. **m**, Pathway enrichment analysis of genes in non-linear expression clusters associated with Aβ density in lecanemab. **n**, LOESS plots of selected genes in LOESS cluster 3. Dark line indicating the LOESS predicted expression and light shading representing standard error of the estimated values. **b-d**, Bar plots display means ± SEM. nAD1, nAD2, and nAD3 each refer to separate samples. **b-f, j-n**, nAD = 3, LCMB = 1. **e-f**, MAST was used to compare expression levels. Covariates included manually annotated region or cortical layer, CDR and gDNA percentage with brain region * sample ID as a random effect (**e**) manually annotated region or cortical layer, CDR, gDNA percentage and brain region with brain region * sample ID as a random effect (**f**). **b-d**, Statistical tests, guided by Shapiro–Wilk and F tests, included t-tests, Mann–Whitney, ANOVA with Tukey’s test, Welch ANOVA with Dunnett’s T3 test, and Kruskal–Wallis with Dunn’s test. **f, m, P**-values are FDR-adjusted using the Benjamini-Hochberg correction. *A2M, alpha-2-macroglobulin; Aβ, amyloid-beta; AD, Alzheimer’s disease; APO, apolipoprotein; APOC1, apolipoprotein C1; APOE, apolipoprotein E; CDR, Cellular Detection Rate; CD68, cluster of differentiation 68; Cort., cortical; Ctx, cortex; CTSB, cathepsin B; DEGs, differentially expressed genes; DESeq2, Differential Expression Analysis for Sequence Count Data (version 2); FCGBP, Fc fragment of IgG binding protein; FDR, False Discovery Rate; gDNA, genomic DNA; GM, gray matter; IBA1, ionized calcium-binding adapter molecule 1; ITGAX, integrin subunit alpha X; L, layer; LCMB, lecanemab; LOESS, locally estimated scatterplot smoothing; MAST, Model-based Analysis of Single-cell Transcriptomics; MSigDB, Molecular Signatures Database; MT, mitochondrial; nAD, non-immunized Alzheimer’s disease; nFeatures, number of features; P-adj, P-value adjusted; SPP1, secreted phosphoprotein 1; ST, spatial transcriptomics*.

**Fig. 4:**
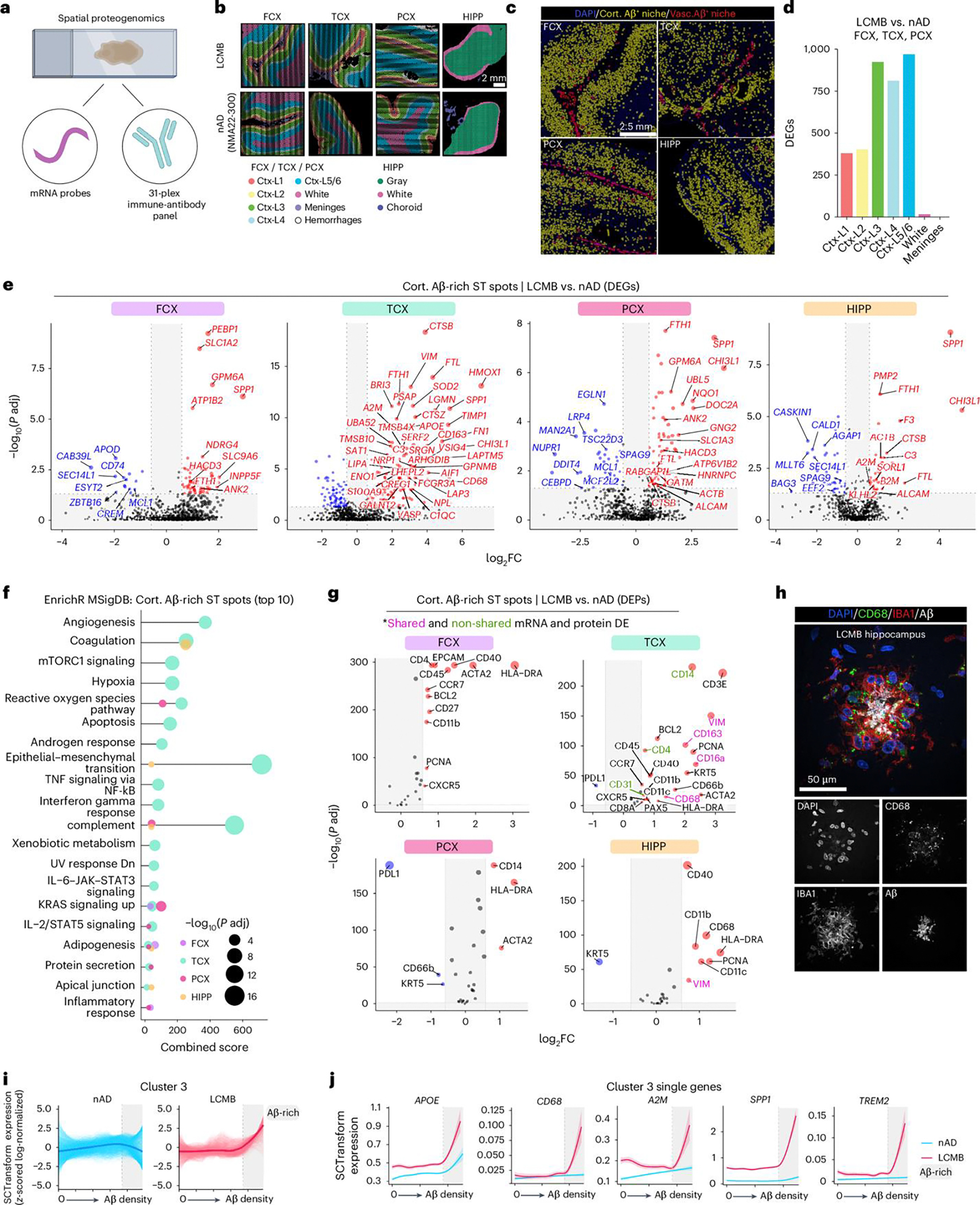
Spatial proteogenomics links the Aβ niche to microglial states. **a**, Proteogenomics allowed for the simultaneous profiling of RNA and protein from lecanemab-treated and nAD controls. Created with BioRender.com. **b**, Manual annotations of brain regions analyzed. **c**, Representative images showing distinction of segmented cortical and vascular Aβ in brain regions from the lecanemab case. **d**, Number of DEGs for each comparison across manually annotated areas. **e**, DEGs from Aβ-rich gray matter ST spots (lecanemab versus nAD) in FCX, TCX, PCX and HIPP. **f**, Top ten pathway enrichment analysis of DEGs in Aβ-rich gray matter ST spots for each brain region (lecanemab versus nAD). **g**, DEPs associated with cortical Aβ ST spots from each brain region (lecanemab versus CAA control), with pink indicating shared DEGs, green indicating no shared DEGs and black indicating low expression levels not meeting DEG criteria. **h**, Confocal images showing CD68^+^IBA1^+^ myeloid cells surrounding Aβ deposits in the HIPP of the lecanemab-treated patient. **i**, LOESS plot of cluster 3 predictions in nAD (left) and lecanemab (right) relative to Aβ density. Dark line represents the mean LOESS predicted expression per group per cluster and single lines indicate LOESS predicted gene expression per group per cluster. **j**, LOESS plots of selected genes in LOESS cluster 3. Dark line indicates the LOESS predicted expression and light shading represents standard error of the estimated values. **d**–**g**,**i**,**j**, nAD = 3; LCMB = 1. DESeq2 (**d**), MAST (**e**) or FindMarkers with a negative binomial model (**g**) was used to compare expression levels. For DESeq2, covariates included brain region, average genes detected and gDNA percentage. In the MAST model, manually annotated region or cortical layer, gDNA percentage and CDR were included as covariates, with brain region and sample ID as a random effect. For FindMarkers, covariates included manually annotated region or cortical layer and CDR. All *P* values were FDR adjusted using Benjamini–Hochberg.

Having established microglial responses to lecanemab using single-cell analysis, we next used spatial proteogenomics on adjacent tissue sections to investigate the immune response at the Aβ niche (Fig. 4a). An 11 × 11-mm ST capture area (14,336 spots) was analyzed, with ST spots annotated using DAPI staining to delineate meninges, cortical layers and white matter, and to exclude hemorrhagic regions (Fig. 4b and Extended Data Fig. 4a). Quality-control metrics revealed variability in the number of expressed genes across nAD brains (Extended Data Fig. 4b) and in mitochondrial read percentages (Extended Data Fig. 4c). Yet, regional comparisons showed no significant differences between the nAD group and the lecanemab case. We distinguished cortical and vascular Aβ, constructed expanded Aβ niches and defined Aβ-rich ST spots (Fig. 4c). Consistent with AN1792, we found reduced cortical Aβ in the superficial layers (I and II; Extended Data Fig. 4d). However, unlike AN1792, differential expression analysis revealed the most DEGs in the deeper cortical layers (III, IV and V/VI; Fig. 4d).

Transcriptomic analysis of cortical Aβ-positive ST spots showed that residual Aβ-rich ST spots in regions with the most clearance (TCX and PCX) were the most dysregulated (Extended Data Fig. 4e). In line with the single-cell analysis of microglia in Fig. 3, Aβ-rich ST spots in the TCX exhibited higher expression of genes related to complement signaling (*C3*, complement C1q C chain (*C1QC*)) and lipid metabolism (*APOE*, lipase A, lysosomal acid type (*LIPA*)); Fig. 4e and Extended Data Fig. 4f). We observed APOE localized to Aβ plaques surrounded by IBA1^+^ myeloid cells in the lecanemab-treated brain (Extended Data Fig. 4g). Additionally, Aβ niches in all regions except the FCX shared upregulated genes involved in lysosomal function and protein degradation (*CTSB*), iron storage (*FTH1*, *FTL*) and extracellular matrix remodeling during inflammation (*CHI3L1*). *A2M* was upregulated in TCX and HIPP Aβ niches. We also observed A2M localized to IBA1^+^ myeloid cells around Aβ deposits (Extended Data Fig. 4h). Notably, we found upregulation of *SPP1* and *FTH1* across Aβ niches in all brain regions. Pathway analysis of Aβ-rich ST spots in the TCX revealed upregulation of complement signaling pathways (Fig. 4f). Intriguingly, adipogenesis pathways increased across all regions, suggesting involvement in lipid metabolism processes (Fig. 4f).

We next evaluated immune responses at the protein level within cortical Aβ niches. We adapted our ST method to include 31 barcoded antibodies targeting immune proteins, which were transferred with RNA probes to generate spatial proteogenomics data. We assessed differentially expressed proteins (DEPs) with or without corresponding DEG transcripts (Fig. 4g). Among DEPs, HLA class II histocompatibility antigen, DR alpha chain (HLA-DRA) was upregulated in Aβ-rich ST spots across all brain regions (Fig. 4g). Proteins linked to the microglial phagocytic response, such as CD11c and CD68, were upregulated in the TCX and HIPP. IHC revealed numerous CD68^+^ lysosomal structures within IBA1^+^ microglia surrounding Aβ deposits in the HIPP (Fig. 4h and Extended Data Fig. 4i). Interestingly, the immune-inhibitory receptor ligand programmed cell death 1 ligand 1 (PD-L1) was reduced in regions with the highest Aβ clearance (TCX, PCX).

We used LOESS to assess nonlinear expression changes within Aβ-rich ST spots in the lecanemab-treated brain (Extended Data Fig. 4j,k). We identified 11 clusters (Extended Data Fig. 4l), with cluster 3 being most associated with immune pathways, including high complement and IL-2–STAT5 signaling (Extended Data Fig. 4m). Cluster 3 was notably upregulated in ST spots with the highest Aβ content in the lecanemab case, and included previously identified genes such as *A2M*, *APOE*, *APOC1*, *CTSB*, *CD68*, *FCGBP*, *ITGAX*, *SPP1* and *TREM2* (Fig. 4i,j and Extended Data Fig. 4n). In summary, proteogenomic analysis of the Aβ niche identified microglial states linked to Aβ clearance in a patient with AD who was treated with lecanemab.

### Shared microglial response drives Aβ clearance after immunization

**Extended Data Fig. 5:**
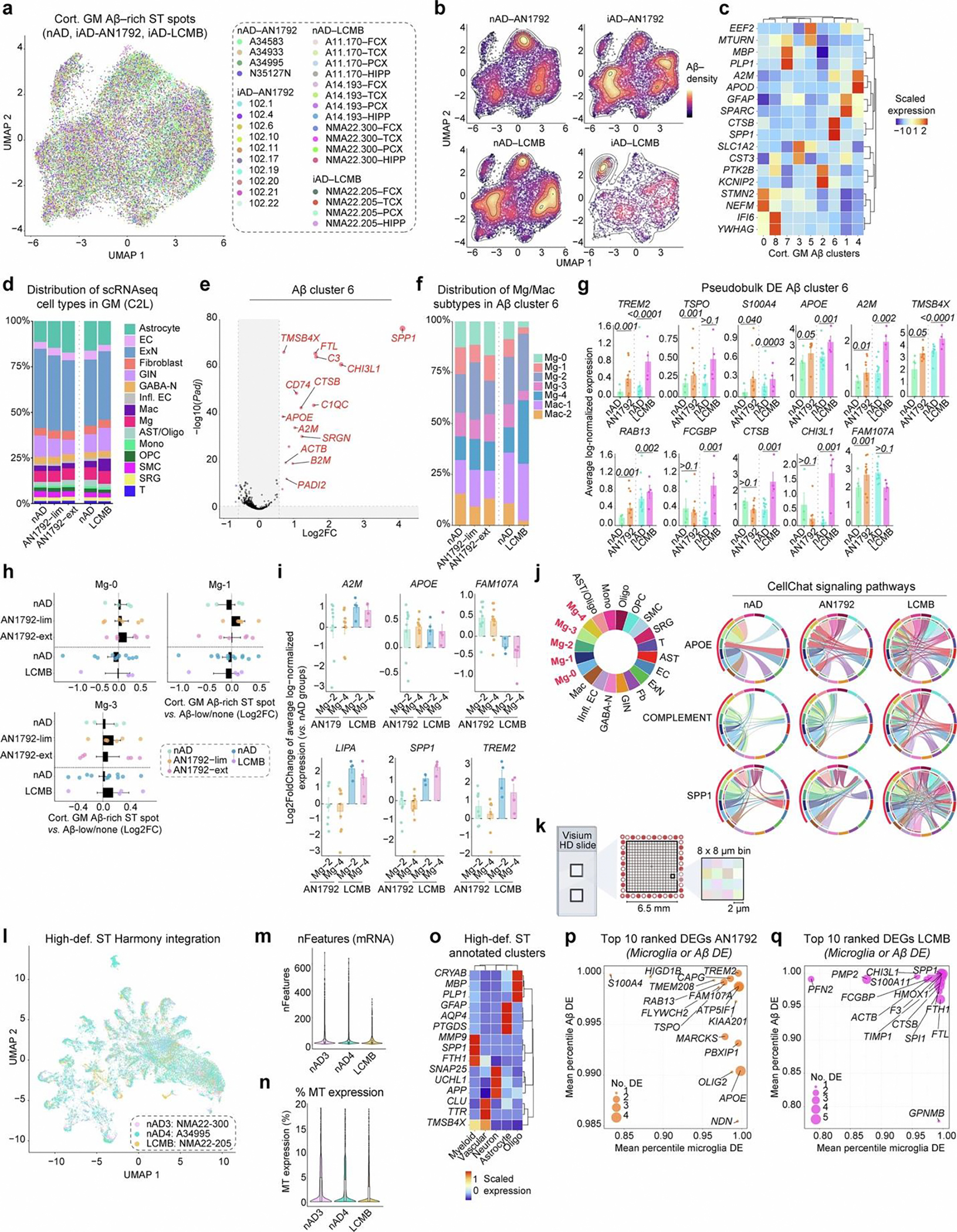
Shared microglial response drives Aβ clearance after immunization. **a**, UMAP showing cortical Aβ-rich ST spots based on gene and protein expression, colored by brain region and donor. **b**, UMAP density plots for each group. **c**, Top two marker genes for each cortical Aβ-rich cluster. **d**, Bar plots showing C2L predictions of scRNA-seq cell types proportionally in the gray matter per group. **e**, Volcano plot showing DEGs distinguishing cortical Aβ-rich cluster 6 from all other cortical Aβ-rich clusters. **f**, Bar plots showing C2L predictions of scRNA-seq microglia and macrophage subtypes proportionally in Aβ-rich cluster 6 per group. **g**, Bar graphs showing pseudobulked *TREM2, TSPO, S100A4, APOE, A2M, TMSB4X, RAB13, FCGBP*, *CTSB, CHI3L1*, and *FAM107A* expression in Aβ-rich cluster 6. Error bars indicate SEM. P-values are from DESeq2. **h**, Bar plots showing log2 fold-change in predicted abundance of deconvoluted scRNA-seq microglia types in Aβ-rich ST spots versus the rest in AN1792, nAD, and the lecanemab case. **i**, Bar plots showing log2 fold-change in pseudobulked expression of *A2M, APOE, FAM107A, LIPA, SPP1*, and *TREM2* in Aβ-associated Mg2-enriched and Mg4-enriched ST spots compared to the nAD control group for AN1792 and the lecanemab case. **j**, Chord plots showing inferred CellChat cell-cell communication of APOE, complement, and SPP1 signaling pathways among different scRNA-seq cell types. The width of the chords reflects the strength of interaction or communication probability, with thicker chords indicating stronger signaling. **k**, Visium HD ST method. Created using BioRender.com. **l**, UMAP showing annotated binned nuclei from the high-definition ST assay, colored by donor. **m**, Number of features (genes) per binned nuclei in high-definition ST data per donor. **n**, Percentage of mitochondrial genes per binned nuclei in high-definition ST data per donor. **o**, Top three marker genes for overarching cell types annotated in the high-definition ST data. **p-q**, Top 10 upregulated response DEGs in microglia or Aβ DE ranked by their average percentile in Aβ (Y-axis) and microglia (X-axis) DE: **o**, in AN1792; **r**, in lecanemab. **g, h-i**, Bar plots display means ± SEM. **m-n**, Violin plots showing the data range and median. Points represent individual cells. nAD3 and nAD4 refer to separate samples. **a-c, e-f**, nAD-AN1792 = 4, iAD-lim = 6; iAD-ext = 4; nAD-LCMB = 3; LCMB = 1. **d**, nAD-AN1792 = 6, iAD-lim = 6; iAD-ext = 7; nAD-LCMB = 3; LCMB = 1. **g**, nAD-AN1792 = 4; iAD = 10; nAD-LCMB = 3; LCMB = 1. **h**, nAD-AN1792 = 4; iAD-lim = 6; iAD-ext = 6; nAD-LCMB = 3; LCMB = 1. i, iAD = 9 (Mg-2), 10 (Mg-4); LCMB = 1. **j**, iAD = 5; nAD = 3; LCMB = 1. **l-o**, nAD = 2; LCMB = 1. **g**, DESeq2 was used to compare expression levels. Covariates included sex, age, average genes detected, gDNA percentage (AN1792 vs. nAD); average genes detected, brain region, gDNA percentage (LCMB vs. nAD). **h**. Statistical tests, guided by Shapiro–Wilk and F tests, included t-tests, Mann–Whitney, ANOVA with Tukey’s test, Welch ANOVA with Dunnett’s T3 test, and Kruskal–Wallis with Dunn’s test. All P-values are FDR-adjusted using the Benjamini-Hochberg correction. *A2M, alpha-2-macroglobulin; Aβ, amyloid-beta; AD, Alzheimer’s disease; APOC1, apolipoprotein C1; APOE, apolipoprotein E; CD68, cluster of differentiation 68; Cort., cortical; Ctx, cortex; CTSB, cathepsin B; DEGs, differentially expressed genes; DESeq2, Differential Expression Analysis for Sequence Count Data (version 2); FDR, False Discovery Rate; FCGBP, Fc fragment of IgG binding protein; GM, gray matter; HD, high-definition; IBA1, ionized calcium-binding adapter molecule 1; ITGAX, integrin subunit alpha X; L, layer; LCMB, lecanemab; LOESS, locally estimated scatterplot smoothing; MSigDB, Molecular Signatures Database; MT, mitochondrial; nAD, non-immunized Alzheimer’s disease; nFeatures, number of features; P-adj, P-value adjusted; SPP1, secreted phosphoprotein 1; ST, spatial transcriptomics*.

**Fig. 5:**
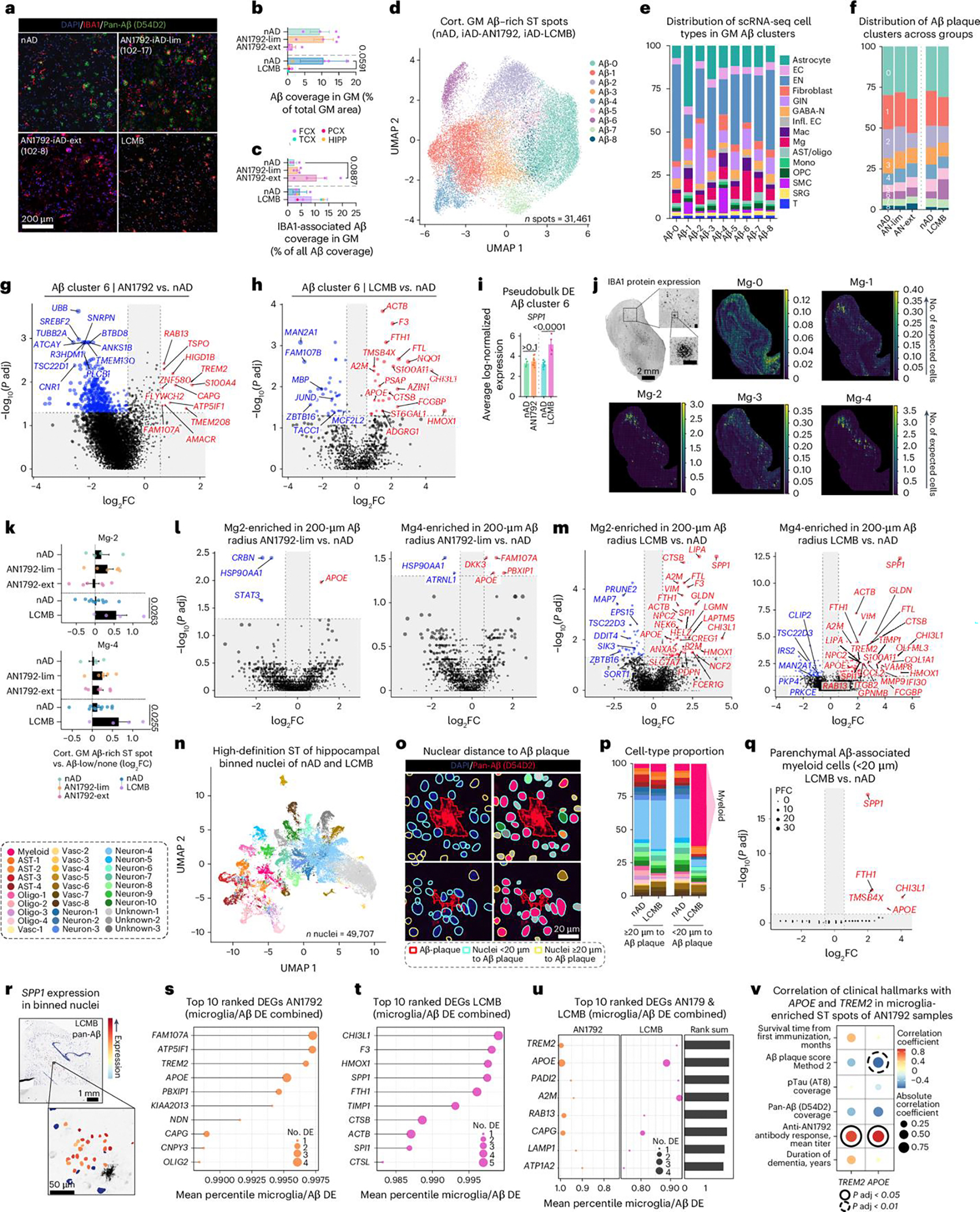
Shared microglial response drives Aβ clearance after immunization. **a**, Confocal images showing pan-Aβ and IBA1 in FCX brain regions of nAD, AN1792-lim, AN1792-ext and lecanemab-treated patients. **b**, Percentage of cortical Aβ coverage in cortical and hippocampal regions of AN1792, nAD and the lecanemab case. **c**, Percentage of cortical Aβ covered by IBA1 in cortical and hippocampal regions of AN1792, nAD and the lecanemab case. **d**, Clustering of Aβ-rich cortical gray matter spots based on gene expression. **e**, C2L predictions of scRNA-seq cell types in different Aβ plaque clusters. **f**, Percentages of Aβ-rich clusters in AN1792, nAD and the lecanemab case. **g**,**h**, DEGs in Aβ-rich cluster 6: AN1792 versus nAD (**g**); lecanemab versus nAD (**h**). **i**, Pseudobulked *SPP1* expression in Aβ-rich cluster 6. Error bars indicate the s.e.m. *P* values are from DESeq2. **j**, Spatial plots showing the abundance of deconvoluted scRNA-seq microglia types; scale bar, 100 μm. **k**, log_2_ fold change in predicted abundance of deconvoluted scRNA-seq microglia types in Aβ-rich ST spots versus the rest in AN1792, nAD and the lecanemab case. **l**,**m**, DEGs from Mg-2-enriched and Mg-4-enriched Aβ-associated ST spots: AN1792 versus nAD (**l**); lecanemab versus nAD (**m**). **n**, UMAP showing annotated binned nuclei from a high-definition ST assay. **o**, Spatial plots indicating the distance of nuclei to D54D2-stained Aβ plaques (left) and their annotations (right). **p**, Percentage of each cell type in the high-definition ST assay at ≥20 μm and <20 μm from Aβ plaques in nAD and the lecanemab case. **q**, DEGs from myeloid nuclei within <20 μm of Aβ plaques (lecanemab versus nAD). CDR is included as a covariate in the MAST model. **r**, Spatial plots showing *SPP1* expression in binned nuclei around Aβ plaques in the lecanemab HIPP. **s**,**t**, Top ten upregulated response DEGs ranked by the average percentile across microglia and Aβ differential expression in AN1792 (**s**) and lecanemab (**t**). **u**, Top ten combined response genes to AN1792 and lecanemab by summing average percentiles of gene ranks. **v**, Covariate-adjusted Spearman correlation between *TREM2* and *APOE* expression in microglia-enriched gray matter ST spots from AN1792 patients and clinical hallmarks. **b**,**c**,**i**,**k**, Bar plots display means ± s.e.m. **b**,**c**, nAD-AN1792 = 3; iAD-lim = 4; iAD-ext = 4; nAD-LCMB = 3; LCMB = 1. **d**–**f**, nAD-AN1792 = 4; iAD-lim = 6; iAD-ext = 4; nAD-LCMB = 3; LCMB = 1. **g**–**i**, nAD-AN1792 = 4; iAD = 10; nAD-LCMB = 3; LCMB = 1. **k**, nAD-AN1792 = 4; iAD-lim = 6; iAD-ext = 6; nAD-LCMB = 3; LCMB = 1. **l**,**m**, nAD-AN1792 = 4; iAD-lim = 6; nAD-LCMB = 3; LCMB = 1. **n**,**p**–**q**, nAD = 2; LCMB = 1. **v**, iAD = 13. **g**,**h**,**l**,**m**,**q**, MAST was used to compare expression levels. Covariates included sex, age, CDR and gDNA percentage with sample ID as a random effect (**g** and **l**); brain region, CDR and gDNA percentage with brain region and sample ID as a random effect (**h** and **m**); CDR (**q**). **i**, DESeq2 was used to compare expression levels. Covariates included sex, age, average genes detected, gDNA percentage (AN1792 versus nAD); average genes detected, brain region and gDNA percentage (lecanemab versus nAD). **v**, Covariates included sex, age, average genes detected and gDNA percentage. **g**–**i**,**l**,**m**,**q**,**v**, *P* values were FDR adjusted using Benjamini–Hochberg. **b**,**c**,**k**, Statistical tests, guided by Shapiro–Wilk and *F* tests, included *t*-tests, Mann–Whitney tests, analysis of variance (ANOVA) with Tukey’s test, Welch’s ANOVA with Dunnett’s T3 test and Kruskal–Wallis with Dunn’s test. AN1792-ext, AN1792 immunized with extensive Aβ clearance; AN1792-lim, AN1792 immunized with limited Aβ clearance; DE, differential expression; ECs, endothelial cells; Mono, monocytes.

To identify common and distinct microglial responses to Aβ after active and passive immunization, we integrated analyses of all tissues. We quantified Aβ coverage in the gray matter, confirming a decrease in coverage associated with immunization (Fig. 5a, b). Additionally, we observed increased myeloid recruitment to Aβ (Fig. 5c). To examine cellular responses to Aβ, we integrated and clustered cortical gray matter Aβ-rich ST spots (Fig. 5d and Extended Data Fig. 5a,b). This yielded nine distinct Aβ niche clusters based on gene expression (Fig. 5e and Extended Data Fig. 5c). We hypothesized that these differences were driven by distinct cellular microenvironments and tested this by using C2L to predict cell-type abundances from our integrated scRNA-seq atlas (Extended Data Fig. 5d). The cortical Aβ-6 cluster, enriched in microglia, was most prominent in the lecanemab sample, followed by a lesser increase in AN1792 samples (Fig. 5f). This cluster was defined by expression of *A2M*, *APOE*, *C1QC*, *C3*, *SPP1* and others (Extended Data Fig. 5e). Thus, the cortical Aβ-6 cluster likely represents Aβ-rich ST spots with recruited myeloid cells. In AN1792 samples with limited Aβ clearance and in lecanemab-treated brain regions, this cluster showed higher microglia abundance of Mg-2 and Mg-4 compared to nAD controls (Extended Data Fig. 5f).

Differential expression analysis of Aβ-6 ST spots between AN1792 and nAD samples identified upregulation of *FAM107A*, *RAB13*, *TREM2* and others in AN1792 samples (Fig. 5g and Extended Data Fig. 5g). In lecanemab-treated ST spots, we observed upregulation of *A2M*, *APOE* and others (Fig. 5h and Extended Data Fig. 5g). *SPP1* lacked zero counts, making the MAST hurdle model unsuitable. Using DESeq2, we revealed that *SPP1* was highly upregulated in lecanemab-treated cortical Aβ-6 ST spots (Fig. 5i). We plotted previously identified microglial subtypes in Aβ-rich ST spots, finding enrichment of Mg-2 and Mg-4 microglia subtypes in Aβ niches of lecanemab-treated brain areas (Fig. 5j,k and Extended Data Fig. 5h). Gene expression analysis of Aβ-associated Mg-2 and Mg-4 ST spots showed increased *APOE* and *FAM107A* in AN1792 samples (Fig. 5l) and upregulated *APOE*, *LIPA*, *SPP1* and *TREM2* among lysosomal function and iron metabolism genes in lecanemab-treated regions (Fig. 5m). Pseudobulked fold changes showed that *FAM107A* is uniquely increased in Mg-2 and Mg-4 Aβ-associated ST spots in AN1792 samples, while *SPP1* and *LIPA* are associated only with lecanemab treatment (Extended Data Fig. 5i). Notably, *APOE* and *TREM2* increased after both treatments (Extended Data Fig. 5i). Using CellChat^45^, we mapped cell-to-cell signaling related to APOE, complement and SPP1 pathways, identifying increased microglial signaling via complement and SPP1 pathways in the lecanemab-treated brain, and elevated APOE signaling in both lecanemab and AN1792 samples (Extended Data Fig. 5j).

To achieve single-cell resolution, we applied high-definition ST to the HIPP of the lecanemab-treated brain and nAD controls (Extended Data Fig. 5k). Nuclei were segmented^46^, clustered and annotated by top markers (Fig. 5n and Extended Data Fig. 5k,o). We mapped nuclei to Aβ plaques using immunofluorescence staining (Fig. 5o). Myeloid cells, putative microglia, were overrepresented within 20 μm of Aβ plaques in the lecanemab-treated brain but not in nAD controls (Fig. 5o,p). Differential expression analysis confirmed increased expression of *SPP1*, *APOE* and others in microglia near Aβ plaques after lecanemab treatment (Fig. 5q). *SPP1* expression was localized to nuclei around Aβ (Fig. 5r). These data validate many of the lower-resolution ST findings throughout the study.

Finally, to identify common and distinct gene expression changes in microglia and at the Aβ plaque niche from AN1792-treated (Figs. 1m, 2e and 5g,l) and lecanemab-treated (Extended Data Fig. 4f and Figs. 3i and 5h,m) brains, we ranked genes by probabilistic fold change (PFC) and assigned percentile ranks. In AN1792 samples, *FAM107A* was the top response gene, followed by ATP synthase inhibitory factor subunit 1 (*ATP5IF1*), *TREM2* and *APOE* (Fig. 5s and Extended Data Fig. 5p). In lecanemab-treated brain areas, *CHI3L1*, *F3*, *HMOX1* and *SPP1* were the top induced genes (Fig. 5t and Extended Data Fig. 5q). Notably, *TREM2* and *APOE* emerged as common responsive genes in both treatments (Fig. 5u). Our analysis highlights both distinct (*FAM107A*, *SPP1*) and common (*APOE*, *TREM2*) microglial response genes related to active and passive Aβ immunization.

We correlated *TREM2* and *APOE* expression with clinical data for AN1792 patients, finding a positive correlation between AN1792 antibody titer and *TREM2*/*APOE* expression in microglia-enriched ST spots (Fig. 5v). There was also a trend toward a negative correlation between *APOE* expression and Aβ plaque score assessed throughout the neocortex using a standardized method^16^. This shows that the expression levels of microglial *APOE* and *TREM2* were directly associated with the immunization response and Aβ clearance. Altogether, our findings delineate the microglial response mediating Aβ clearance in AD brains immunized against Aβ.

## Discussion

This study defines the microglial response to Aβ immunization in patients with AD. We detected upregulation of *APOE* and *TREM2* in microglia of both actively and passively immunized brains. Notably, side effects from passive immunization are more common in *APOE* ε4 carriers^9,14,47^, and antibodies targeting TREM2 have been explored as therapeutic strategies for AD^48,49^. Our findings indicate that APOE and TREM2 play crucial roles in microglial responses to Aβ immunization, suggesting the microglial response influences both efficacy and risk of adverse effects.

We observed a decrease in the expression of genes related to protein folding and cellular stress in microglia of iAD brains, indicating a more favorable cerebral environment after immunization. Further, we observed elevated expression of neuroprotective genes, including *FGFR3*, in microglia-enriched ST spots of extensively cleared brains. FGFR3–FGF2 signaling may facilitate beneficial neuronal–microglial communication and support neuronal health in cleared Aβ regions. Our data also indicate a metabolic shift in microglia during Aβ immunization. In brains with extensive Aβ clearance, we found increased oxidative phosphorylation and reduced glycolysis in microglia. This suggests that active immunization can mitigate chronic neuroinflammation in AD and reestablish homeostasis in brains with extensive Aβ clearance.

To examine passive immunization, we studied a patient with *APOE* ε4 homozygous AD who was treated with lecanemab. Our scRNA-seq analysis revealed increased presence of two microglial subtypes in regions with the highest Aβ clearance. Both subtypes spatially associated with Aβ plaques and expressed DAM markers (for example, *APOE* and *TREM2*) and showed elevated complement signaling. Yet, these subtypes differed in their expression of homeostatic markers, as well as their expression of *AXL*, *C3* and *CD74*. Complement signaling, particularly C3, plays a role in Aβ clearance by aiding Aβ recognition and phagocytosis by microglia^50^.

Comparative analysis of active and passive immunization revealed that residual Aβ niches and microglia in the lecanemab brain uniquely upregulated *CHI3L1* and *SPP1* signaling, while AN1792 treatment upregulated *FAM107A* and *ATP5IF1*. Lecanemab-treated brain areas also showed increased expression of lysosomal and protein degradation genes. Both treatments increased *APOE* and *TREM2* expression and reduced HSP-coding genes and stress-responsive microglial states.

Importantly, we found that *APOE* and *TREM2* expression in microglia-enriched ST spots correlated positively with anti-AN1792 antibody titer and trended toward a negative correlation with Aβ plaque load. This suggests that a more robust and sustained immune response to vaccination is linked to long-term microglial *APOE* and *TREM2* expression and enhanced Aβ clearance. These findings support the hypothesis that microglial APOE and TREM2 are instrumental in sustained Aβ clearance following immunization.

Few studies have reported postmortem cases of lecanemab-treated patients^18,51^. Our study includes one rare case with multifocal intracerebral hemorrhage. Therefore, future studies with larger sample sizes and longitudinal designs are needed to validate and extend our findings related to passive immunization. Animal models can further dissect the mechanistic role of our key microglial markers in Aβ clearance. Additionally, the effects of *APOE* genotype and sex on immune responses to Aβ immunization warrant further investigation. Despite the limited sample size, our analysis of rare human samples provides valuable insights into the molecular mechanisms driving Aβ clearance and suggest potential targets for enhancing immunotherapy efficacy.

In summary, we reveal distinct microglial phenotypes linked to Aβ immunization in the AD brain. These findings provide new insights into the microglial mechanisms underlying Aβ clearance and lay the foundation for refining next-generation immunotherapies in AD.

## Online content

Any methods, additional references, Nature Portfolio reporting summaries, source data, extended data, supplementary information, acknowledgements, peer review information; details of author contributions and competing interests; and statements of data and code availability are available at https://doi.org/10.1038/s41591-025-03574-1.

## Methods

### Human tissue samples

#### AN1792 study cohort.

Clinical and neuropathologic follow-up of patients with AD enrolled in the Elan Pharmaceuticals phase I trial of AN1792 was previously reported^3,10–12^. FCX tissue was available from 22 patients with iAD, of whom 16 had a neuropathologic diagnosis of AD. The remaining 6 patients had a different cause of dementia and were excluded from further analysis. Notably, 3 of the 16 AD brains (cases 2, 3 and 9) were omitted due to low RNA quality scores, leaving a final cohort of 13 iAD samples. One patient (case 1) required imaging in life, which demonstrated features of meningoencephalitis^10^ and neuroradiological features consistent with the later-defined amyloid related imaging abnormalities (ARIA) edema^16^. With inadequate numbers of postmortem placebo-treated samples from the original AN1792 trial, frontal cortices of 6 nAD cases and 6 NND cases were used as controls. Cases were matched as closely as possible for age at death. In total, postmortem FFPE frontal cortical samples of 13 iAD (mean age of death, 79.97 years; range, 63–89 years), 6 nAD controls (mean age of death, 79.60 years; range, 65–89 years) and 6 NND controls (mean age of death, 74.93 years; range, 63–82) were included in the active immunization analysis. All nAD cases and 4 NND FCX samples were sourced from Stanford Alzheimer’s Disease Research Center. Two additional NND samples were sourced from Northwestern Pathology. Relevant clinical and demographic information of iAD, nAD and NND cases are listed in Extended Data Table 1. Tissue blocks were cut into 5-μm sections and stored at 4 °C until further use.

#### Lecanemab and nAD controls.

Clinical and neuropathologic findings of a 65-year-old *APOE* ε4/ε4 female patient with early cognitive decline treated with lecanemab were previously reported^17,18^. In the open-label phase, the patient received three intravenous lecanemab infusions—each 2 weeks apart. Four days after the final dose, the patient developed stroke-like symptoms, received tissue plasminogen activator and suffered fatal intracerebral hemorrhages. Consent was obtained to perform full-body postmortem examination and subsequent reporting of the neuropathologic findings related to her receiving anti-Aβ. Multiple foci of histiocytic/microglial reaction to parenchymal amyloid plaques were noted. According to NIA-AA 2012 consensus guidelines^52^, the AD neuropathologic changes would be categorized as ‘high’. FFPE tissue blocks from the FCX, TCX, PCX and HIPP of both donors were sectioned into 5-μm slices and stored at 4 °C. The nAD control samples (mean age at death, 69.3 years; range, 62–82 years) were matched for parenchymal AD pathology (high), vascular AD pathology and *APOE* ε4/ε4 genotype. Notably, one nAD donor also had magnetic resonance imaging-positive microbleeds on gradient echo sequences. Relevant clinical and demographic information of lecanemab and nAD cases are listed in Extended Data Table 2.

### Ethics declarations for human tissues

#### AN1792 tissue.

This study was conducted in compliance with all relevant ethical guidelines and was approved by BRAIN UK (UK Brain Archive Information Network) under REC reference 19/SC/0217.

#### ROSMAP data.

All participants in the Religious Orders Study and Rush Memory and Aging Project (ROSMAP) enrolled without known dementia and agreed to detailed clinical evaluation and brain donation at death^53^. Both studies were approved by an Institutional Review Board (IRB) of Rush University Medical Center (ROS IRB no. L91020181, MAP IRB no. L86121802). Both studies were conducted according to the principles expressed in the Declaration of Helsinki. Each participant signed an informed consent, an Anatomic Gift Act and an RADC Repository consent (IRB no. L99032481) allowing their data and biospecimens to be repurposed.

#### Lecanemab tissue and nAD controls.

Consent was obtained to perform postmortem examination and subsequent reporting of the neuropathologic findings related to the patient receiving lecanemab. The study of de-identified nAD tissue was approved by the IRB of Northwestern University (exempt IRB no. 00219860).

### DNA collection and genotyping

gDNA was extracted from residual brain material on glass slides following ST workflow, using the QIAamp DNA FFPE Tissue Kit (catalog no. 56404, Qiagen), with deparaffinization steps omitted as they were completed during the ST protocol. DNA was isolated from all nAD and NND samples, as well as AN1792 samples 102–19 and 102–20, which lacked *APOE* genotype information. Positive controls were included to validate genotyping. Quality and concentration of extracted DNA were assessed to ensure suitability for genotyping. *APOE* genotyping for the single-nucleotide polymorphisms (SNPs) rs429358 and rs7412 was conducted at the University of Illinois at Chicago Genomics Research Core using the BioMark HD Real-Time PCR system (Fluidigm) and SNP Type assays (rs429358: C_3084793_20; rs7412: C_904973_10; Thermo Fisher). Genotyping was performed according to the manufacturer’s protocol, with each SNP assayed using TaqMan SNP Genotyping Assays (Applied Biosystems). Genotype calling was conducted with the SNP Genotyping Analysis software (Fluidigm) using default analysis parameters: a confidence threshold of 65, global normalization and *k*-means clustering. PCR cycle 35 was used for SNP calling, and each sample was analyzed in three technical replicates. No template controls were incorporated into vacant inlets as negative controls. Allele calls were determined based on fluorescence signals from FAM and VIC probes, and each *APOE* haplotype was assigned by combining the alleles of rs429358 and rs7412, resulting in the following classifications: ε2 (T/T), ε3 (T/C) and ε4 (C/C).

### ST

FFPE samples were deparaffinized, stained with H&E and decrosslinked according to the Visium CytAssist Spatial Gene Expression for FFPE protocol (CG000520 rev. B, 10x Genomics). H&E-stained tissues were imaged on an EVOS M7000 Imaging System (AMF7000, Thermo Fisher Scientific) using a ×20 objective (0.45 NA, AMEP4982, Thermo Fisher Scientific). Immediately after decrosslinking, libraries were prepared according to the user guide for Visium CytAssist Spatial Gene Expression Reagent Kits (CG000495, rev. E, 10x Genomics). Final libraries were sequenced by the NUSeq Core at Northwestern University Feinberg School of Medicine using the Illumina NovaSeq 6000 or Illumina NovaSeq X Plus platforms to the recommended depth of 25,000 reads per tissue-covered ST spot. The Space Ranger pipeline version 2.0.0., referencing the GRCh38 human genome (GENCODE v32/Ensembl 98), and Visium Transcriptome Probe Set v2.0 (10x Genomics) were used to process FASTQ files. ST spots were annotated in the Loupe Browser (10x Genomics) using the high-resolution images to delineate meninges, cortical layers and white matter.

### Spatial proteogenomics

FFPE samples were deparaffinized, decrosslinked and stained with a combination of DAPI (1:100 dilution; 62248; Thermo Fisher), rabbit anti-IBA1 (1:250 dilution; 019–19741; WAKO) and mouse anti-pan-Aβ (1:250 dilution; clone 4G8; 800708; BioLegend). Notably, we used TrueBlack Plus Lipofuscin Autofluorescence Quencher (23014; Biotum) according to the manufacturer’s instructions. Tissues were imaged on an EVOS M7000 Imaging System (AMF7000, Thermo Fisher Scientific) using a ×20 objective (0.45 NA, AMEP4982, Thermo Fisher Scientific, or an Olympus Lucplanfl N ×20/0.45 Ph1 UIS2 Collar Fn22). After imaging, spatial gene and protein expression libraries were immediately prepared according to the user guide for Visium CytAssist Spatial Gene and Protein Expression Reagent Kits (CG000494; rev. B; 10x Genomics). We used the Visium Human Transcriptome Probe Set version 2.0 for RNA transcript detection, along with the Human FFPE Immune Profiling Panel, which includes a 35-plex CytAssist Panel of antibodies, both intracellular and extracellular, sourced from BioLegend and Abcam for protein detection. This panel also comprises four isotype controls. Final libraries were sequenced as detailed above for ST. The targeted sequencing depth was 25,000 reads per tissue-covered ST spot for gene expression libraries, and 5,000 reads per tissue-covered ST spot for protein expression libraries, as recommended. The Space Ranger pipeline version 2.1.1., referencing the GRCh38 human genome (GENCODE v32/Ensembl 98), and Visium Transcriptome Probe Set v2.0 (10x Genomics) were used to process FASTQ files. ST spots were annotated in the Loupe Browser (10x Genomics) using the high-resolution images to delineate meninges, cortical layers and white matter, and to exclude hemorrhagic regions.

### High-definition ST

FFPE samples were deparaffinized, decrosslinked and stained with DAPI, rabbit anti-pan-Aβ (1:500 dilution; clone D54D2, 8243, Cell Signaling Technology), goat anti-IBA1 (1:100 dilution; ab5076, Abcam) and mouse anti-phospho-Tau (1:250 dilution; MN1020, Thermo Fisher) according to the Visium HD FFPE Tissue Preparation Handbook (CG000684 rev. A, 10x Genomics). Lipofuscin autofluorescence was quenched with TrueBlack Lipofuscin Quencher. Stained tissues were imaged on an EVOS M7000 Imaging System (AMF7000, Thermo Fisher Scientific) using a ×20 objective (Olympus Lucplanfl N ×20/0.45 Ph1 UIS2 Collar Fn22). Immediately following decrosslinking, libraries were prepared per the Visium HD Spatial Gene Expression Reagent Kits user guide (CG000685 rev. B, 10x Genomics). Final libraries were sequenced by the NUSeq Core at Northwestern University Feinberg School of Medicine on an Illumina NovaSeq X Plus platform to a target depth of 275 million reads per fully covered capture area. FASTQ files were processed using the Space Ranger pipeline version 3.0.0, referencing the GRCh38 human genome (GENCODE v32/Ensembl 98) and Visium Transcriptome Probe Set v2.0 (10x Genomics).

### scRNA-seq

For each sample, 1–2 consecutive FFPE scrolls of 25 μm were prepared and processed according to the Isolation of Cells from FFPE Tissue Sections for Chromium Fixed RNA Profiling protocol (CG000632, 10x Genomics). After deparaffinization and dissociation by pestle, single cells were hybridized with barcoded probes overnight. GEM generation and library construction were performed as outlined in the Chromium Fixed RNA Profiling Reagent Kits for Multiplexed Samples manual (CG000527; rev. E; 10x Genomics). The first batch included four brain regions—FCX, TCX, PCX and HIPP—from one nAD control (NMA22–300) and one lecanemab-treated sample (NMA22–205). The second batch contained the same regions from two additional nAD controls (A14–193 and A11–170). The third batch contained FCX samples from ten AN1792 samples (102–1, 102–7, 102–8, 102–11, 102–15, 102–16, 102–17, 102–19, 102–21 and 102–22). To enhance cell yield per tissue, samples were split across 2 barcodes per pool, totaling 16 barcodes. Limited cell numbers in samples 102–7, 102–8, 102–11 and 102–21 restricted them to a single barcode each. We targeted approximately 8,000 cells per barcode, aiming for a total of 16,000 cells per tissue per pool. For batches one and three, two pools were generated, targeting 32,000 cells across both pools for samples with dual barcodes. Cell counts were taken at several stages to ensure consistent pooling, using a DAPI stain (1:2,000 dilution; 62248; Thermo Fisher) and imaged with an EVOS M7000 Imaging System (AMF7000, Thermo Fisher Scientific) using a ×4 objective lens (0.13 NA, AMEP4980, Thermo Fisher Scientific). The final libraries were indexed and pooled, and then sequenced together by the NUSeq Core at Northwestern University Feinberg School of Medicine on an Illumina NovaSeq X Plus sequencer, aiming for approximately 25,000 reads per cell. Demultiplexed FASTQ files were processed using the Cell Ranger pipeline version 7.2.0, referencing the GRCh38 human genome (GENCODE v32/Ensembl 98) and the Visium Transcriptome Probe Set v2.0 (10x Genomics).

### IHC

#### DAB hematoxylin staining.

Consecutive sections from the ST data, spaced 5–10 μm apart, were used to stain for pan-Aβ. FFPE sections were heated at 60 °C for 1 h, followed by incubation in xylenes and a graded ethanol series. Antigen retrieval was performed at 95 °C for 30 min in either citrate buffer pH 6.0 (64142–08, Electron Microscopy Sciences) or Tris-EDTA pH 9.0 (AB93684, Abcam). Slides were blocked using 10% normal goat serum (ab7481, Abcam) in PBS with 0.03% Triton-X (21568–2500, Acros Organics) for up to 4 h. The sections were then incubated overnight at 4 °C with the primary antibody for pan-Aβ (1:100 dilution; clone D54D2, 8243, Cell Signaling) and subsequently with goat anti-rabbit horseradish peroxidase (1:200 dilution; P0448, Agilent Technologies) for 1 h at room temperature. Sections were then treated with diluted DAB chromogen (K3468, Dako) for 20 min at room temperature. Hematoxylin (51275, Sigma-Aldrich) was used for counterstaining before the sections were dehydrated and mounted with Cytoseal (8312–4, Epredia). Nonadjacent serial sections were also stained for phosphorylated tau using the AT8 antibody (1:500 dilution; Thermo Fisher Scientific, MN1020) using this protocol.

### Immunofluorescence

FFPE sections were placed in an oven for 1 h at 60 °C before deparaffinization in xylenes and rehydration with a series of graded ethanol. Antigen retrieval was performed at 95 °C for 30 min in citrate buffer (pH 6.0; 64142–08, Electron Microscopy Sciences) or Tris-EDTA buffer (pH 9.0; Ab93684, Abcam). Slides were blocked using 10% of normal donkey serum (017–000-121, Jackson ImmunoResearch; Ab7475, Abcam) in PBS with 0.03% Triton-X (21568–2500, Acros Organics) for up to 4 h. Sections were then incubated with primary antibodies (Extended Data Table 3) overnight at 4 °C, followed by a 1-h incubation in Alexa Fluor-labeled secondary antibodies (1:400 dilution) at room temperature. Primary antibodies used included goat anti-IBA1 (1:150 dilution; ab5076, Abcam), rabbit anti-Aβ (1:1,000 dilution; clone D54D2, 8243, Cell Signaling), mouse anti-CD68 (1:400 dilution; clone KP1, ab955, Abcam), rabbit anti-IBA1 (1:400 dilution; 019–19741, WAKO), goat anti-APOE (1:500 dilution; ab947, Sigma-Aldrich), mouse anti-Aβ (1:1,000 dilution; clone D3D2N, 15126, Cell Signaling), rabbit anti-TMS1/ASC (1:400 dilution; clone RM1049, ab309497, Abcam), mouse anti-Aβ (1:250 dilution; clone 4G8, 800708, BioLegend), rabbit anti-A2M (1:500 dilution; clone EPR4432, ab109422, Abcam), rabbit anti-APOC1 (1:300 dilution; clone EPR16813, ab198288, Abcam) and rabbit anti-SPP1 (1:300 dilution; ab8448, Abcam). Immunofluorescence-stained slides were counterstained for DNA using DAPI (1:5,000 dilution; 62248, Thermo Fisher), followed by quenching of autofluorescence with TruBlack Plus (1:40 dilution; 23014, Biotium) in PBS. Slides were mounted using ProLong Gold Antifade Mountant (P36934; Fisher Scientific).

### Imaging analysis

#### Imaging and processing of pan-Aβ DAB stains in consecutive ST images.

Tissue imaging was performed with a TissueGnostics slide scanner. The acquired images were processed using Fiji software (National Institutes of Health (NIH)). Briefly, deconvolution was applied to the images for the hematoxylin and DAB staining. Manual thresholds were set for Aβ reactivity using the DAB stain by a researcher blinded to sample identification. The derived binary signal was further cleaned by removing small particles. Aβ coverage in the gray matter was determined by calculating the ratio of Aβ deposits in the gray matter to the total area of the gray matter per sample. To construct the expanded Aβ niches, the binary Aβ signal was artificially extended by 100 μm from its original boundary, with a gradual decrease in signal intensity noted every 20 μm. Subsequently, the images were aligned to the CytAssist image using the Loupe browser (version 7.0.1, 10x Genomics) and further integrated with the spatial RNA data using Space Ranger version 2.1.1. Importantly, ST spots with unreliable Aβ staining, arising from technical issues or tissue anomalies such as folds or holes, were omitted from subsequent Aβ niche analyses. Additionally, vascular Aβ ST spots were excluded from any further analyses.

#### Imaging and processing of phosphorylated Tau (AT8) DAB stains.

Tissue imaging for AT8-stained slides was performed at ×20 magnification using an automated slide scanner microscope (Olympus VS110, Olympus America) at the Biomedical Imaging Unit, Faculty of Medicine, University of Southampton. The acquired images were processed using Fiji software (NIH). Briefly, deconvolution was applied to separate the hematoxylin and DAB signals. Manual thresholds were set for AT8 reactivity in the DAB channel by a researcher blinded to sample identification. The resulting binary signal was refined by removing small particles. AT8 coverage in the gray matter was quantified as the ratio of AT8-positive area to the total gray matter area per sample.

#### Processing of immunofluorescence images for spatial proteogenomics.

High-resolution imaging in the spatial proteogenomic workflow was conducted using Fiji. The DAPI channel was auto-thresholded using the Li vector, with subsequent removal of entities larger than ~1 cm and application of the watershed function to separate binary nuclear masks. The IBA1 channel processing involved dividing the image into a 25 × 25 grid, applying Bleach Correction via Histogram Matching to each segment, reassembling the image and using the RollingBall algorithm (radius, 2.8 μm) to reduce background noise. Both IBA1 and Aβ channels underwent manual thresholding, conducted by a researcher blinded to sample identification. After binarization, channels were subjected to two rounds of dilation and erosion, followed by a filtering step to remove oversized objects in Aβ and IBA1, targeting noise reduction. The binarized IBA1 masks were then utilized to refine the bleach-corrected IBA1 channel. This refinement was achieved by overlaying the binarized IBA1 mask onto the bleach-corrected IBA1 channel. In this process, only the regions within the confines of the binarized mask were retained, while areas outside the mask were cleared.

Vascular and cortical Aβ were identified in the high-resolution images from the spatial proteogenomic workflow using the LabKit machine learning tool^54^ within Fiji. Each sample was analyzed with a unique classifier to generate a vascular Aβ probability map. This map was initially enhanced with despeckling and Gaussian blur (*σ* = 4) to improve smoothness, followed by triple dilation and erosion and filtering to exclude small particles. The probability maps were then manually thresholded by a researcher blinded to sample identification. Observations of vascular Aβ missed by the automated process but detected upon visual inspection were carefully annotated and included. The vascular Aβ binary signal was dilated twice before being extracted from the processed Aβ channel, leaving the residual signal to be identified as cortical Aβ. Expanded Aβ niches were subsequently delineated as described above. For nAD controls A14–193 and A11–170, vascular Aβ was manually annotated instead of using LabKit. Importantly, ST spots exhibiting unreliable Aβ staining, whether due to technical complications or the presence of tissue folds or holes, were excluded from further analyses related to the Aβ niche.

#### Aβ coverage and IBA1 colocalization.

The coverage of cortical or total Aβ within the gray matter was determined by calculating the percentage of the area covered by the binarized cortical or total Aβ mask in the gray matter to the total area of the gray matter for each brain region per donor. To evaluate the association between IBA1^+^ cells and cortical or total Aβ, the area where cortical Aβ and IBA1 colocalized was divided by the total area of cortical or total Aβ present in the gray matter.

### Data preprocessing, quality control and integration

#### AN1792 RNA for ST.

Seurat objects were initialized for each sample with Space Ranger’s filtered feature barcode matrices using Load10X_Spatial. ST spots with extremely high or low unique molecular identifier (UMI) counts or extremely low feature counts were removed on a per-sample basis. Outermost ST slide spots, ST spots with at least 20% mitochondrial expression and ST spots that were not covering tissue were removed. Raw counts were independently normalized using log-normalization and SCTransform normalization, with SCTransform models fit per sample. Depending on the assay, raw counts, log-normalized data or SCTransformed data were utilized.

#### scRNA-seq.

Cell Ranger’s filtered feature barcode matrices for each sample in each pool were corrected for background contamination using SoupX42. Low-quality cells were removed before doublet identification, using a sample-specific and pool-specific minimum UMI threshold of three median absolute deviations below the median and a minimum feature threshold of two median absolute deviations below the median. Additionally, cells exhibiting mitochondrial gene expression above 20% were removed. Doublet identification was performed with DoubletFinder utilizing ten principal components, a pN setting of 0.25 and a pool-specific predicted doublet rate determined based on the average number of cells loaded per probe barcode, with a 0.4% undetectable multiplet rate for 825 cells loaded per barcode as per the Chromium Fixed RNA Profiling Reagent Kits for Multiplexed Samples manual (CG000527; rev. E; 10x Genomics). Doublets were removed, and samples from AN1792 donors 102–1, 102–16, 102–17, 102–19 and 102–22 were retained, while donors 102–7, 102–8, 102–11 and 102–21 were excluded due to high contamination fractions, low UMI counts or high mitochondrial expression. The remaining raw counts were further processed with SCTransform while adjusting for mitochondrial gene expression, with SCTransform models fit for each sample in each pool. Fifty principal components were derived from the SCTransform-processed data, which was then harmonized across both pools and samples using the IntegrateLayers function with the HarmonyIntegration approach. Lastly, UMAP visualization was constructed from 30 integrated features.

#### Lecanemab RNA for ST.

Seurat objects were initialized for each sample with Space Ranger’s filtered feature barcode matrices using Load10X_Spatial. ST spots with extremely high or low UMI counts or extremely low feature counts were removed on a per-sample basis. ST spots with at least 20% mitochondrial expression were removed for FCX, TCX and PCX and ST spots with at least 30% mitochondrial expression were removed for HIPP. ST spots not located on cortical or hippocampal tissue were excluded. Outermost ST slide spots, and ST spots with zero protein expression were removed. Raw counts were independently normalized using log-normalization and SCTransform normalization, with SCTransform models fit per sample. Depending on the assay, raw counts, log-normalized data or SCTransformed data were utilized.

#### Lecanemab spatial protein analysis.

For each sample, Seurat objects were created from Space Ranger’s filtered feature barcode matrices, which included isotype-normalized counts, via the Load10X_Spatial function. Isotype control antibodies were excluded from further analysis.

#### High-definition ST analysis.

Nuclei segmentation was performed using StarDist^46^, with expression data from Space Ranger’s 2 × 2-μm filtered feature barcode matrices assigned to segmented nuclei. Nuclei with fewer than ten UMI counts or over 20% mitochondrial expression were excluded. Raw counts were transformed with SCTransform, with SCTransform models fit per sample. Fifty principal components were derived from the SCTransform-processed data. Integration across samples was achieved with IntegrateLayers using the HarmonyIntegration method, and a UMAP was generated using 30 integrated features.

### snRNA-seq integration and reference generation

A snRNA-seq dataset^33,34^ from ROSMAP^53^ was used to generate a reference atlas for ST data. We initially identified 5,000 feature genes per batch in Seurat using FindVariableFeatures. From this pool of genes, we selected 5,000 common feature genes across all datasets for anchor identification using the FastFindAnchors function from the FastIntegration package^55^. The resulting batch-corrected values were subsequently used for downstream analyses, including principal component analysis, UMAP and clustering. For annotation of broad cell types, we utilized the feature genes identified during the integration stage. Subsequently, within each broad cell type, we reselected feature genes and conducted similar analyses to delineate detailed subtypes. Major cell types used were astrocytes, endothelial cells, stromal cells, immune cells, oligodendrocytes, oligodendrocyte precursor cells, interneurons, ENs, fibroblasts, pericytes and smooth muscle cells. We then randomly downsampled 2,000 cells per cell type, or included all cells for categories with fewer than 2,000 cells, resulting in a reference dataset of 34,695 cells.

### Cell-type annotation

#### scRNA-seq.

Clustering was conducted in Seurat by first applying the FindNeighbors function with 30 integrated features, followed by FindClusters at multiple resolutions, with a final resolution of 1 used to define initial clusters. To identify immune subtypes, the data were subsetted to clusters expressing immune markers. Data from different cohorts was merged, and raw counts were transformed using SCTransform, while adjusting for mitochondrial gene expression, with SCTransform models fit per cohort. Fifty principal components were generated from the SCTransform data, and integration across cohorts was performed using IntegrateLayers with the CCA integration method. Clustering was refined by reapplying FindNeighbors and FindClusters with 30 integrated features, defining immune clusters at a resolution of 0.35. A UMAP was generated using the 30 integrated features.

#### Aβ niches.

Data from all cohorts were subsetted to include cortical Aβ-rich ST spots in gray matter. Raw counts were transformed using SCTransform, with SCTransform models fit per sample and 50 principal components were generated from SCTransform data. Integration across samples was conducted using IntegrateLayers with the HarmonyIntegration method. A UMAP was generated with 30 integrated features, followed by clustering with Seurat’s FindNeighbors and FindClusters functions across various resolutions. Final clusters were defined at a resolution of 0.4.

#### High-definition ST.

Clustering of Visium HD data was conducted in Seurat using FindNeighbors with 30 integrated features, followed by FindClusters across multiple resolutions, with final clusters defined at a resolution of 0.2.

### ST deconvolution

For spatial deconvolution, we utilized the C2L (v0.1.3) package^32^. Gene filtering on the reference data was conducted using the filter_genes function, with parameters set to cell_count_cutoff = 5, cell_percentage_cutoff2 = 0.03 and nonz_mean_cutoff = 1.12. The batch_key parameter was configured as sequencing batch for the snRNA-seq atlas and sample ID for the scRNA-seq atlas, with each sample ID corresponding to a specific brain region (for example, the same donor had a distinct sample ID for each brain region). The reference regression model was trained for 500 epochs for the reference atlas and 750 epochs for our in-house-created scRNA-seq immunization atlas to stabilize the evidence lower bound loss. The ROSMAP FCX snRNA-seq atlas was used to deconvolute ST spots across all regions, while our in-house-created scRNA-seq immunization atlas was used to deconvolute ST spots in gray matter. The proportion of genes expressed per ST spot (CDR) was calculated from raw counts and standardized. ST spots were deconvoluted using the resulting reference signatures, with standardized CDR as a continuous covariate and sample ID as the batch key in the C2L model. The model was trained in batches of 2,500 ST spots over 1,000 epochs to stabilize the evidence lower bound loss. To account for technical variability in RNA detection sensitivity, the detection_alpha parameter was set to 20, and the N_cells_per_location parameter was set to 7 based on manual cell counts of several ST spots. The 5% quantile of the posterior distribution was computed directly and used for downstream analysis.

### Defining cell-type-enriched ST spots

To identify ST spots enriched for specific cell types, we applied cell-type-specific region restrictions and thresholds to C2L predictions, with enrichment defined separately for each sample. For cell types from the snRNA-seq reference atlas, ST spots enriched for fibroblasts, pericytes, peripheral immune cells, smooth muscle cells and endothelial cells were annotated when C2L predictions for a given ST spot were in the top 1% of gray and white matter or the top 5% of meningeal ST spots. Microglia and astrocytes were considered enriched in the top 5% of gray or the top 5% of white matter. Interneuron enrichment was defined in the top 5% of gray matter, while MYO16 ENs were enriched in the top 1% of gray matter. Oligodendrocyte precursor cells were enriched in the top 5% of both gray and white matter, and oligodendrocytes were considered enriched in the top 30% of white matter. Enrichment for layer-specific neurons was determined within the relevant cortical layers: L2/3 ENs in the top 10% of layers II–III; L4 ENs in the top 50% of layer IV; L4/5 ENs in the top 15% of layers IV–VI; and L5, L5/6, L5/6 CCa and L5/6 CCb ENs in the top 5% of layers V–VI. Layer I was excluded from enrichment analysis for all cell types in the snRNA-seq atlas. For microglia clusters from our in-house scRNA-seq immunization atlas, enrichment was defined by a sample-specific C2L prediction threshold set at three standard deviations above the mean in gray matter ST spots.

### Definition of Aβ enrichment groups

ST spots were classified as Aβ-rich if the coverage within the expanded Aβ niche, indicated by barcode fluorescence intensity, exceeded a threshold of 183. The Aβ niche was defined to include Aβ-rich ST spots along with their first-order and second-order spatial neighbors in gray and white matter, based on array coordinates, covering an approximate radius of 200 μm. ST spots containing CAA pathology, as well as ST spots immediately adjacent to those, were excluded from the cortical Aβ niche. ST spots with unreliable Aβ staining, arising from technical issues or tissue anomalies such as folds or holes, were omitted from Aβ niche analyses.

### DEG analysis

To identify DEGs across various regions of interest within our datasets, we used two distinct differential expression techniques: DESeq2 (ref. 56) and MAST^29^. Each approach was adapted to suit the specific characteristics and requirements of the comparison, considering the nature of the data (pseudobulk for DESeq2 and single-cell method for MAST) and the level at which covariates were standardized (sample level for DESeq2 and ST spot or cell level for MAST). Covariate selection was guided by variance partition analysis, which identified gDNA as the primary driver of variance after the experimental group. Additionally, we accounted for the number of genes expressed in a subset of ST spots or cells to control for differences in quality and sequencing depth. Sex and age were included due to their known effects on immune responses in AD. Additionally, manually annotated regions or cortical layer annotations per ST spot were incorporated where applicable to address sampling variability across anatomical areas.

#### DESeq2.

DESeq2 was initiated by subsetting the data for ST spots within the region of interest (ROI). Continuous covariates at the sample level, including age, average nFeatures within subsampled ST spots and gDNA percentage, were standardized within each ROI. We then filtered out genes not expressed in at least 1% of either comparison group based on raw counts, excluding genes starting with RPS, RPL, MT or HB. Pseudobulk data were created by summing raw counts by donor to facilitate a more robust differential expression analysis. The DESeq2 analysis was conducted with the inclusion of covariates such as sex, age, average features within subsampled ST spots and gDNA percentage, all of which were standardized, if continuous. The DESeq function was run with fitType = ’local’ to estimate dispersions using local regression, the results function was run with independentFiltering = FALSE to include low-expression genes in statistical testing, and LFC shrinkage was performed by running lfcShrink with type = ’apeglm’. *P* values were adjusted using the Benjamini–Hochberg method. DEG significance thresholds were set at an adjusted *P* value of 0.05 and a log_2_ fold change of ±log_2_(1.5).

#### MAST.

For MAST, data were subsetted to include only the ST spots or cells of interest. Sample-specific and brain region-specific downsampling was applied to ensure that no single sample contributed more than 50% of ST spots or cells within a comparison group, that the fold difference in total ST spots or cells between comparison groups did not exceed three, and that each group contained no more than 3,000 ST spots or cells. Continuous covariates at the ST spot level, including age, the CDR from recorrected SCT data and gDNA percentage per sample, were standardized within each subset of ST spots or cells. We applied PrepSCTFindMarkers on the ROI, which recorrects SCTransform counts to normalize sequencing depth across samples. SCT data (log1p-transformed SCT counts) were then extracted from the Seurat object. Genes prefixed with RPS, RPL, MT or HB were excluded, and additional filtering was performed based on percentage expression within comparison groups. Genes were tested if they were expressed in 1% of both groups and in 10% of either group using SCT expression data, except for Visium HD data, where genes were tested if they were expressed in 1% of either group. The log_2_ fold change between comparison groups for the remaining genes was calculated using the Seurat FoldChange function. MAST was run with covariates such as sex, age, CDR, gDNA percentage, brain region and manually annotated regions or cortical layers, all of which were standardized if continuous. Sample ID, or brain region and sample ID, was included as a random effect for comparisons involving multiple samples. MAST hurdle *P* values were adjusted using the Benjamini–Hochberg method. LFC from the prior calculation was appended to the results, with significance thresholds for differential expression set at an adjusted *P* value of 0.05 and a LFC of ±log_2_(1.5).

### DEP analysis

Data were first subset for the ROI, and CDR (calculated based on isotype-normalized counts) was standardized within the ROI. A negative binomial generalized linear model was used through Seurat’s FindMarkers function for differential expression analysis on isotype-normalized counts, setting min.pct to 0.01, logfc.threshold to -Inf, and standardized CDR and manually annotated regions or cortical layers as latent variables. Raw *P* values were adjusted using the Benjamini–Hochberg method, and proteins were considered significant DEPs with adjusted *P* value less than 0.05 and magnitude of average log fold change greater than log_2_(1.5).

### Marker expression defining cell types or Aβ niche types

SCTransform-corrected counts were recorrected using PrepSCTFindMarkers, with log-transformed (log1p) corrected counts utilized in analyses. To delineate general cluster markers, the FindMarkers function facilitated the identification of positive marker genes through a ‘one-versus-many’ comparative approach, testing genes expressed in more than 25% of the cluster of interest (set to 1% for Visium HD), setting only.pos to TRUE and using the default Wilcoxon rank-sum test. Genes prefixed with RPS, RPL, MT or HB were excluded from testing. Marker gene selection was based on Benjamini–Hochberg-adjusted *P* values below 0.05. For the specific analysis of positive and negative markers within cortical Aβ niche cluster 6, FindMarkers was used to compare cluster 6 against all others, allowing for both positive and negative marker detection (only.pos = FALSE), with min.pct set to 0.1 and logfc.threshold set to -Inf, using the default Wilcoxon test. Genes prefixed with RPS, RPL, MT or HB were excluded. Marker genes were deemed significant if they presented an adjusted *P* value under 0.05 and an average LFC exceeding log_2_(1.5).

### Gene-set enrichment analysis

#### Human MSigDB.

Gene lists were analyzed using the enrichR package^57^ with the hallmark gene-set collection from the Human MSigDB. For lists containing specifically downregulated genes, combined scores were negated. A significance threshold was set at a Benjamini–Hochberg-adjusted *P* value of 0.05.

#### Microglia states.

Signed probability fold change was calculated for each gene as the product of the negative logarithm of the adjusted *P* value and the log_2_ fold change. Enrichment for human microglial activation states^34,41^ was assessed using the fgsea package, with probability fold change as the ranking metric. Custom gene sets associated with various microglial activation states were compiled from the supplementary materials provided in the referenced studies. Normalized enrichment scores were calculated, and significance was determined through permutation testing, with *P* values adjusted using the Benjamini–Hochberg method. A threshold of 0.05 was applied for adjusted *P* values, with no specific cutoff for the magnitude of NES values.

### LOESS trajectory analysis

LOESS was used to identify nonlinear patterns of gene expression across the Aβ niche in gray matter separately for each group. SCTransform counts were readjusted through PrepSCTFindMarkers, with the logarithm of one plus the corrected counts (log1p) serving as the basis for our analysis. Predictions were generated for all genes in the SCT assay. A LOESS regression of span 0.75 was fit to each gene within each group using the LOESS function of the R stats package. Predicted expression values were standardized within each group. The predicted expression trajectories across the Aβ niche were then subdivided into clusters, using hierarchical clustering through the hclust function in the R stats package.

### CellChat

Cells were grouped by broad cell type or microglia subtype, and data were subset by treatment group. SCTransform-corrected counts were recorrected using PrepSCTFindMarkers, with log-transformed (log1p)-corrected counts utilized in the analysis. ComputeCommunProb was run with the population.size parameter set to TRUE in order to account for the proportion of cells in each cell group, with a 10% truncated mean used to calculate average gene expression per cell group.

### Quantification and statistical analysis

Statistical analyses were conducted primarily using R (version 4.2.3) and GraphPad Prism (version 10.2.1). In GraphPad Prism, normality and variance equality were assessed using the Shapiro–Wilk and *F* tests, respectively, to guide test selection. For two-group comparisons, we applied unpaired two-tailed Student’s *t*-tests (with or without Welch’s correction for unequal variances) or Mann–Whitney tests. For comparisons involving more than two groups, we used one-way ANOVA with Tukey’s multiple-comparisons test, Welch’s ANOVA with Dunnett’s T3 multiple-comparisons test, or a Kruskal–Wallis test with Dunn’s multiple-comparisons test. Relative abundances, including scRNA-seq-derived cell types, microglia clusters and Aβ niche clusters, were compared using paired *t*-tests. For all analyses, statistical significance was defined as *P* value < 0.05, with multiple testing correction applied when appropriate, using an adjusted *P* value < 0.05.

### ShinyCell

Our ShinyCell app enables users to explore scRNA-seq gene expression patterns on a UMAP, conduct comparative analyses of gene expression across different groups using violin/box plots and access supplementary built-in analytical tools.

## Extended Data

**Extended Data Table 1 |:** AN1792 cohort demographics

											AN1792 Specifics							
Case	Brain Region	Group ID	Neuropathological diagnosis	Origin	AD Neuropathological change	Age at death (years)	Sex (m/f)	Additional findings	APOE4 allele copy number	Braak stages	AN1792 dose (μg)	Number of injections	Anti-AN1792 mean titre	Histological evidence of plaque removal Method 1	Aβ plaque score Method 2 (CERAD-adapted)	Duration of dementia (years)	Last known dementia status	Survival time from first immunization (months)

A18.148	FCX	NND	NND	Northwestern Pathology	NA	82	f	--	1	--	--	--	--		--	--	--	--
A34285	FCX	NND	NND	Stanford ADRC	NA	75	m	--	N/A	--	--	--	--		--	--	--	--
A34291	FCX	NND	NND	Stanford ADRC	NA	63	m	Mild-artherosclerosis	1	--	--	--	--		--	--	--	--
A34717	FCX	NND	NND	Stanford ADRC	NA	79	m	--	1	--	--	--	--		--	--	--	--
A34992	FCX	NND	NND	Stanford ADRC	NA	68	m	--	1	--	--	--	--		--	--	--	--
AX21.92	FCX	NND	NND	Northwestern Pathology	NA	82	f	--	1	--	--	--	--		--	--	--	--

A34583	FCX	nAD	AD	Stanford ADRC	A3-B3-C2	89	m	Hippocampal-CA1-TDP-42-type-A	1	--	--	--	--		--	--	--	--
A34644	FCX	nAD	AD	Stanford ADRC	A3-B3-C2	80	f	Mild-non-AD-tauopathy	1	--	--	--	--		--	--	--	--
A34933	FCX	nAD	AD	Stanford ADRC	A3-B3-C3	80	m	--	1	--	--	--	--		--	--	--	--
A34995	FCX	nAD	AD	Stanford ADRC	A3-B3-C3	65	f	--	1	--	--	--	--		--	--	--	--
A35038	FCX	nAD	AD	Stanford ADRC	A3-B3-C2	88	m	--	1	--	--	--	--		--	--	--	--
N35127N	FCX	nAD	AD	Stanford ADRC	A3-B3-C2	76	m	--	1	--	--	--	--		--	--	--	--

102.10	FCX	iAD-lim (AN1792)	AD	AN1792, Elan Pharmaceuticals	A3-B3-C3	88	f	None	0	4/5	50	7	137	+	2.9	11	Moderate	86
102.11	FCX	iAD-lim (AN1792)	AD	AN1792, Elan Pharmaceuticals	A3-B3-C2	88	m	Severe-CAA	1	4/5	50	8	142	+	2.3	12	Moderate	94
102.17	FCX	iAD-lim (AN1792)	AD	AN1792, Elan Pharmaceuticals	A3-B3-C3	86	f	Small-old-right-parietal-infarct-microvascular-lesions-entorhinal-cortex	2	4/5	50	NA	0	+	2.7	13	Severe	141
102.19	FCX	iAD-lim (AN1792)	AD	AN1792, Elan Pharmaceuticals	A3-B3-C3	75	f	Severe-CAA	2	4/5	50	8	221	-	2.1	19	Severe	162
102.22	FCX	iAD-lim (AN1792)	AD	AN1792, Elan Pharmaceuticals	A3-B3-C3	74	m	Extensive-capCAA	2	4/5	225	NA	1313	+	2.3	18	Severe	184
102.4	FCX	iAD-lim (AN1792)	AD	AN1792, Elan Pharmaceuticals	A3-B3-C3	71	f	Severe-CAA	0	4/5	225	8	4072	+	1.5	10	Severe	44

102.1	FCX	iAD-ext (AN1792)	AD	AN1792, Elan Pharmaceuticals	A3-B3-C3	74	f	Numerous-cortical-microvascular-lesions-related-to-severe-CAA-meningoencephalitis	1	4/5	50	5	119	++	0.7	6	Severe	20
102.16	FCX	iAD-ext (AN1792)	AD	AN1792, Elan Pharmaceuticals	A3-B3-C0	89	f	Extensive-capCAA-small-acute-infarct-left-basal-ganglia	1	4/5	225	6	142	+++	0.4	15	Severe	111
102.20	FCX	iAD-ext (AN1792)	AD	AN1792, Elan Pharmaceuticals	A3-B3-C2	82	m	Extensive-capCAA	1	4/5	225	3	430	+++	1	17	Severe	166
102.21	FCX	iAD-ext (AN1792)	AD	AN1792, Elan Pharmaceuticals	A3-B3-C1	87	f	Extensive-capCAA	1	4/5	50	8	3045	+++	0.6	18	Severe	173
102.6	FCX	iAD-ext (AN1792)	AD	AN1792, Elan Pharmaceuticals	A3-B3-C3	81	m	Severe-CAA	1	4/5	50	8	1707	++	1.6	7	Severe	57
102.7	FCX	iAD-ext (AN1792)	AD	AN1792, Elan Pharmaceuticals	A1-B3-C0	82	m	--	1	4/5	50	8	4374	+++	0.05	6	Severe	60
102.8	FCX	iAD-ext (AN1792)	AD	AN1792, Elan Pharmaceuticals	A1-B3-C1	63	m	--	1	4/5	50	8	6470	+++	0.5	10	Severe	64

Aβ, amyloid-beta; AD, Alzheimer’s disease; ADRC, Alzheimer’s Disease Research Center; APOE, apolipoprotein E; CAA, cerebral amyloid angiopathy; capCAA, capillary cerebral amyloid angiopathy; CERAD, Consortium to Establish a Registry for Alzheimer’s Disease, classification of neuritic plaque pathology in AD; CVA, cerebrovascular accident; DVT, deep vein thrombosis; FTLD-TDP, frontotemporal lobar degeneration with TAR DNA-binding protein 43 pathology; LBD, Lewy body disease, neocortical (diffuse); PSP, progressive supranuclear palsy; VBI, vascular brain injury; FCX, frontal cortex; iAD-lim, immunized with limited Aβ clearance; iAD-ext, immunized with extensive Aβ clearance; m/f, male/female; NA, not available/applicable; nAD, non-immunized Alzheimer’s disease; NND, non-neurologic disease; TDP-43, TAR DNA-binding protein 43; Anti-AN1792 mean titre, mean antibody titre against AN1792; Braak stages, classification of tau pathology in AD; APOE allele copy number, number of APOE-ε4 alleles; duration of dementia, duration in years; last known dementia status, severity at last assessment; survival time from first immunization, months from first AN1792 dose to death. Method 1: Analysis of plaque removal was scored semi-quantitatively in frontal, temporal, parietal and occipital neocortex as: very extensive (that is nearly complete clearance of plaques) = +++; intermediate (that is multiple and/or extensive plaque-free foci each involving a >1 cm length of cortical ribbon) = ++; very limited (that is single and/or small plaque-free foci each involving each involving a <1 cm length of cortical ribbon) = +; no evidence of plaque removal = –. Method 2: Quantification of amyloid-β plaques in coronal hemisphere sections (score out of 3).

* LBD with marked capillary angiopathy but no plaques, possibly reflecting clearance of diffuse plaques. Alzheimer’s disease neuropathological change: A = amyloid-β plaque score; B = neurofibrillary tangle stage; C = neuritic plaque score according to National Institute on Aging-Alzheimer’s Association guidelines (Hyman et al., 2012).

**Extended Data Table 2 |:** Lecanemab cohort demographics

Spatial proteogenomics and single-cell RNA sequencing									

Case	Brain Region(s)*	Group ID	Figure ID	Neuropathological diagnosis	Origin	Age at death (years)	Sex (m/f)	Additional notes	APOE4 allele copy number

NMA22-205	FCX, TCX, PCX, HIPP	LCMB	LCMB	AD	Northwestern Pathology	65	f	Participated in phase III trial of lecanemab, received three lecanemab infusions over the course of five weeks	2
A14-193	FCX, TCX, PCX, HIPP	nAD	nAD1	AD	Northwestern Pathology	64	f	Developed acute ischemic stroke-like symptoms shortly after the final lecanemab dose and passed away days later.	2
A11-170	FCX, TCX, PCX, HIPP	nAD	nAD2	AD	Northwestern Pathology	66	f		2
NMA22-300	FCX, TCX, PCX, HIPP	nAD	nAD3	AD	Northwestern Pathology	82	m		2

High-definition spatial transcriptomics									

Case	Brain Region(s)*	Group ID	Figure ID	Neuropathological diagnosis	Origin	Age at death (years)	Sex (m/f)	Additional notes	APOE4 allele copy number

NMA22-205	HIPP	LCMB	LCMB	AD	Northwestern Pathology	65	f	Participated in phase III trial of lecanemab, received three lecanemab infusions over the course of five weeks	2
NMA22-300	HIPP	aAD	nAD3	AD	Northwestern Pathology	82	m	Developed acute ischemic stroke-like symptoms shortly after the final lecanemab dose and passed away days later	2
A34995	HIPP	aAD	nAD4	AD	Stanford ADRC	65	f		1

* In the metadata, the brain regions are referred to by the following scheme: A indicates nAD sample while B denotes LCMB sample; region 1 is FCX, 3 is TCX, 4 is PCX, and 9 is HIPP. Aβ, amyloid-beta; AD, Alzheimer’s disease; APOE, apolipoprotein E; APOE allele copy number, number of APOE-ε4 alleles; FCX, frontal cortex; HIPP, hippocampus; LCMB, lecanemab; m/f, male/female; NA, not available/applicable; nAD, non-immunized Alzheimer’s disease; PCX, parietal cortex; TCX, temporal cortex; TDP-43, TAR DNA-binding protein 43; Stanford ADRC, Stanford Alzheimer’s Disease Research Center; Age at death, age in years at time of death; Additional notes, extra relevant clinical or procedural information.

**Extended Data Table 3 |:** Antibody information

Antibody information							

Target		Concentration	Secondary		DAPI	Trublack Plus	Antigen Retrieval

Gt anti-APOE [ab947]	Sigma	1:500	Dk anti-Gt647	Invitrogen			
Ms anti-Aβ [D3D2N]	Cell Signaling	1:1000	Dk anti-Ms 488	Invitrogen	1:5000	1:40	Citrate
Rb anti-Iba1 [019-19741]	Wako	1:500	Dk anti-Rb 568	Invitrogen			

Ms anti-CD68 [KP1]	Abeam	1:400	Dk anti-Ms 488	Invitrogen			
Rb anti-Aβ [D5402]	Cell Signaling	1:1000	Dk anti-Rb 647	Invitrogen	1:5000	1:40	Citrate
Gt anli-Iba1 [ab5076]	Abcam	1:150	Dk anti-Gt 568	Invitrogen			

Rb anti-TMS1/ASC [RM1049]	Abcam	1:100	Dk anti-Rb 594	Invitrogen			
Ms anti-N3 [4G8]	BioLegend	1:500	Dk anti-Ms 647	Invitrogen	1:5000	1:40	Citrate
Gt anti-Iba1 [ab5076]	Abcam	1:100	Dk anti-Gt488	Invitrogen			

Rb anti-A2M [EPR4432]	Abcam	1:250	Dk anti-Rb 594	Invitrogen			
Ms anti-Aβ [4G8]	BioLegend	1:500	Dk anti-Ms 647	Invitrogen	1:5000	1:40	Citrate/Tris-EDTA
Gt anti-Iba1 [ab5076]	Abcam	1:100	Dk anti-Gt488	Invitrogen			

Rb anti-APOC1 [EPR16813]	Abcam	1:200	Dk anti-Rb 594	Invitrogen			
Ms anti-Aβ [4G8]	BioLegend	1:500	Dk anti-Ms 647	Invitrogen	1:5000	1:40	Tris-EDTA
Gt anti-Iba1 [ab5076]	Abcam	1:100	Dk anti-Gt488	Invitrogen			

Rb anti-SPP1 [ab8448]	Abcam	1:100	Dk anti-Rb 594	Invitrogen			
Ms anti-Aβ [4G8]	BioLegend	1:500	Dk anti-Ms 647	Invitrogen	1:5000	1:40	Citrate
Gt anli-Iba1 [ab5076]	Abcam	1:100	Dkanti-Gt488	Invilrogen			

## Supplementary Material

vanOlst_PMID40050704_NatMed_2025_SupplReportingSummary

vanOlst_PMID40050704_NatMed_2025_Suppl_SourceDataFig1

vanOlst_PMID40050704_NatMed_2025_Suppl_SourceDataFig2

vanOlst_PMID40050704_NatMed_2025_Suppl_SourceDataFig3

vanOlst_PMID40050704_NatMed_2025_Suppl_SourceDataFig4

vanOlst_PMID40050704_NatMed_2025_Suppl_SourceDataFig5

## Data Availability

Spatial RNA and single-cell RNA-seq data have been deposited at the Gene Expression Omnibus under accession numbers GSE263038, GSE263034, GSE263079 and GSE282928. Any additional information required to reanalyze the data reported in this work paper is available from the corresponding author upon request. Data can be explored and requested through a central hub located at https://sites.google.com/view/adimmunization/home. Source data are provided with this paper.
