# Author Correction: Microglial mechanisms drive amyloid-β clearance in immunized patients with Alzheimer’s disease

**DOI:** 10.1038/s41591-025-03664-0

**Published:** 2025-03-28

**Authors:** Lynn van Olst, Brooke Simonton, Alex J. Edwards, Anne V. Forsyth, Jake Boles, Pouya Jamshidi, Thomas Watson, Nate Shepard, Talia Krainc, Benney MR Argue, Ziyang Zhang, Joshua Kuruvilla, Lily Camp, Mengwei Li, Hang Xu, Jeanette L. Norman, Joshua Cahan, Robert Vassar, Jinmiao Chen, Rudolph J. Castellani, James AR Nicoll, Delphine Boche, David Gate

**Affiliations:** 1 Abrams Research Center on Neurogenomics, Northwestern University Feinberg School of Medicine, Chicago, IL USA; 2 The Ken & Ruth Davee Department of Neurology, Northwestern University Feinberg School of Medicine, Chicago, IL USA; 3 Department of Pathology, Northwestern University Feinberg School of Medicine, Chicago, IL USA; 4 Bioinformatics Institute, Agency for Science, Technology and Research, Singapore, Singapore; 5 Clinical Neurosciences, School of Clinical and Experimental Sciences, Faculty of Medicine, University of Southampton, Southampton, UK; 6 Mesulam Center for Cognitive Neurology and Alzheimer’s Disease, Northwestern University Feinberg School of Medicine, Chicago, IL USA; 7 Centre for Computational Biology and Program in Cancer and Stem Cell Biology, Duke-NUS Medical School, Singapore, Singapore; 8 Immunology Translational Research Program, Department of Microbiology and Immunology, Yong Loo Lin School of Medicine, National University of Singapore, Singapore, Singapore; 9 Department of Cellular Pathology, University Hospital Southampton National Health Service Trust, Southampton, UK

**Keywords:** Neuroimmunology, Molecular neuroscience, Alzheimer's disease

Correction to: *Nature Medicine* 10.1038/s41591-025-03574-1, published online 6 March 2025.

In the version of this article initially published, there were typographical errors in the heading of Fig. 5u, where “AN1792” appeared as “AN179”; in Fig. 5v, where “*P* adj. <0.1” appeared as “*P* adj. <0.01”; and in Extended Data Fig. 5i, top-left *x*-axis label, where “AN1792” appeared as “AN179.” The figures have been updated in the HTML and PDF versions of the article.

