# Supplementary material for: Microglial mechanisms drive amyloid-β clearance in immunized patients with Alzheimer’s disease
Source: Nat Med. Author manuscript; Available in PMC 2025 Jun 26. (PMC12092304; doi:10.1038/s41591-025-03574-1)
Supplement: vanOlst_PMID40050704_NatMed_2025_SupplReportingSummary [file NIHMS2066263-supplement-vanOlst_PMID40050704_NatMed_2025_SupplReportingSummary.pdf]

nature portfolio

Corresponding author(s): David Gate

Last updated by author(s): Nov 11, 2024

Reporting Summary

Nature Portfolio wishes to improve the reproducibility of the work that we publish. This form provides structure for consistency and transparency in reporting. For further information on Nature Portfolio policies, see our Editorial Policies and the Editorial Policy Checklist.

Statistics

For all statistical analyses, confirm that the following items are present in the figure legend, table legend, main text, or Methods section.

n/a	Confirmed	
☐	☒	The exact sample size (*n*) for each experimental group/condition, given as a discrete number and unit of measurement
☐	☒	A statement on whether measurements were taken from distinct samples or whether the same sample was measured repeatedly
☐	☒	The statistical test(s) used AND whether they are one- or two-sided<br>*Only common tests should be described solely by name; describe more complex techniques in the Methods section.*
☐	☒	A description of all covariates tested
☐	☒	A description of any assumptions or corrections, such as tests of normality and adjustment for multiple comparisons
☐	☒	A full description of the statistical parameters including central tendency (e.g. means) or other basic estimates (e.g. regression coefficient) AND variation (e.g. standard deviation) or associated estimates of uncertainty (e.g. confidence intervals)
☐	☒	For null hypothesis testing, the test statistic (e.g. *F*, *t*, *r*) with confidence intervals, effect sizes, degrees of freedom and *P* value noted<br>*Give P values as exact values whenever suitable.*
☐	☒	For Bayesian analysis, information on the choice of priors and Markov chain Monte Carlo settings
☒	☐	For hierarchical and complex designs, identification of the appropriate level for tests and full reporting of outcomes
☐	☒	Estimates of effect sizes (e.g. Cohen's *d*, Pearson's *r*), indicating how they were calculated

Our web collection on statistics for biologists contains articles on many of the points above.

Software and code

Policy information about availability of computer code

Data collection

During the ST workflow, tissues were imaged using an EVOS M7000 Imaging System (AMF7000, ThermoFisher Scientific) with a 20x-objective (0.45NA, AMEP4982, ThermoFisher Scientific, or Olympus Lucplanfl N 20x/0.45 Ph1 UIS2 Collar Fn22). The final spatial transcriptomic libraries were sequenced by the NUSeq Core at Northwestern University Feinberg School of Medicine using either the Illumina NovaSeq 6000 or the NovaSeq X Plus platforms. The sequencing targeted a depth of 25,000 reads per tissue-covered ST spot for gene expression libraries, 5,000 reads per spot for protein expression libraries, and 275 million reads per fully-covered capture area for Visium HD libraries; following recommended guidelines.

In the scRNAseq workflow, cells were imaged using the same EVOS M7000 Imaging System with a 4x objective lens (0.13NA, AMEP4980, ThermoFisher Scientific). The final libraries were indexed, pooled, and sequenced together at the NUSeq Core on an Illumina NovaSeq X Plus sequencer, targeting approximately 25,000 reads per cell.

Consecutive sections stained for pan-Aβ were imaged using a 20x-objective (0.5NA) on a TissueGnostics slide scanner at the Northwestern University Center for Advanced Microscopy.

The sequenced data were processed using 10X Space Ranger software versions 2.0.0 for ST AN1792, 2.1.1 for ST Lecanemab/nAD, and 3.0.0 for Visium HD. Additionally, 10X CellRanger version 7.2.0 was used for scRNAseq.

Data analysis

All code used to generate the figures in this study can be found at https://github.com/gatelabNW/AD_Immunization.

Imaging were analyzed and quantified using FIJI, by a researcher blinded to sample identification. Graphpad Prism version 10.2.1 was used for analyses specific to microscopy measurements and the comparison of relative abundances of scRNAseq-derived cell types and microglia

clusters, and Aβ niche clusters. For quality-control metrics on the sample level, we first applied Shapiro–Wilk test and F tests to evaluate normality and variance equality, informing the selection of appropriate statistical tests. The chosen tests included the unpaired two-tailed Student's t-test, with or without Welch's correction for unequal variance, as needed, and the Mann–Whitney test for non-parametric data. For comparisons of relative abundances (scRNAseq-derived cell-types, microglia clusters, and Aβ niche clusters), a paired t-test was utilized. To compare quality-control metrics (such as the number of features and the percentage of mitochondrial expression) between the lecanemab and nAD ST spots, an unpaired, two-sided Wilcoxon test without a continuity correction was used. Across all analyses, a P-value threshold of less than 0.05 was set to denote statistical significance.

Statistical analyses in R v4.2.3 primarily used the following packages: Seurat v5.1.0, sctransform v0.4.1, SoupX v1.6.2, DoubletFinder v2.0.4, MAST v1.27.1, DESeq2 v1.38.3, CellChat v2.1.2, fgsea v1.24.0, enrichR v3.2, ShinyCell v2.1.0. Analyses in R v4.2.0 used the following packages: FastIntegration v1.0.0. Analyses in python v3.9 primarily used the following packages: cell2location v0.1.3, stardist v0.9.1.

For manuscripts utilizing custom algorithms or software that are central to the research but not yet described in published literature, software must be made available to editors and reviewers. We strongly encourage code deposition in a community repository (e.g. GitHub). See the Nature Portfolio guidelines for submitting code & software for further information.

Data

Policy information about availability of data

All manuscripts must include a data availability statement. This statement should provide the following information, where applicable:

- Accession codes, unique identifiers, or web links for publicly available datasets
- A description of any restrictions on data availability
- For clinical datasets or third party data, please ensure that the statement adheres to our policy

Spatial RNA/protein and single-cell RNA-seq data have been deposited at GEO under accession numbers GSE263034, GSE263038 and GSE263079. Data can be explored and requested through a central hub located at: https://sites.google.com/view/adimmunization/home.

Human research participants

Policy information about studies involving human research participants and Sex and Gender in Research.

Reporting on sex and gender

The NND and nAD subjects were matched as closely as possible to their respective experimental groups (AN1792 and lecanemab) based on age at death and pathology, with additional matching by sex where possible. In the AN1792 group, 7 out of 13 subjects (54%) were female, compared to 2 out of 6 (33%) in the nAD control group and 2 out of 6 (33%) in the NND control group. The lecanemab sample was a 65-year-old woman, with the nAD control group consisting of 2 out of 3 women (67%). To account for variations in age and sex, all analyses included age and sex as covariates. The sex and age of each subject are consistently documented throughout the manuscript.

Population characteristics

AN1792, nAD and NND controls: Post-mortem FFPE frontal cortex samples were obtained from 13 iAD patients (mean age = 79.97 years, range = 63–89) enrolled in the AN1792 phase I trial, following previous clinical and neuropathological reporting. Frontal cortex samples from 6 nAD controls (mean age = 79.60 years, range = 65–89) and 6 NND controls (mean age = 74.93 years, range = 63–82) were included as matched controls due to limited availability of placebo-treated samples. All nAD cases and 4 NND cases were sourced from the Stanford Alzheimer's Disease Research Center, with 2 additional NND samples from Northwestern Pathology. Extended clinical and demographic data for iAD, nAD, and NND cases are provided in Extended Data Table 1.

Lecanemab and nAD controls: The lecanemab-treated case involved a 65-year-old woman with AD and significant CAA pathology, including cortical vasculitis and multifocal hemorrhage, consistent with anti-Aβ treatment effects. FFPE sections from frontal, temporal, parietal, and hippocampal regions were analyzed. Comparative nAD controls (mean age = 69.3 years, range = 62–82) were matched by APOE ε4/ε4 genotype, CAA, and high AD pathology. Extended demographic and clinical information for lecanemab and matched nAD cases are available in Extended Data Table 1.

Recruitment

Brain tissue from AD patients enrolled in the Elan Pharmaceuticals Phase I AN1792 trial was obtained through the University of Southampton, with post-mortem examination consent provided by patients or their caretakers. Control samples for the neuropathologically confirmed AD (nAD) cases and four non-neurologic disease (NND) cases were sourced from the Stanford Alzheimer's Disease Research Center, while two additional NND samples were provided by Northwestern Pathology.

The lecanemab-treated brain sample was obtained from a 65-year-old woman with AD through Northwestern Pathology, with consent for a full-body post-mortem examination and reporting of neuropathological findings related to anti-Aβ treatment. For comparative purposes, her brain was evaluated against APOE ε4/ε4 genotype-matched pathological nAD controls provided by Northwestern Pathology.

Ethics oversight

Collection of brain tissue was approved by the Institutional Review Board of each university.

Note that full information on the approval of the study protocol must also be provided in the manuscript.

Field-specific reporting

Please select the one below that is the best fit for your research. If you are not sure, read the appropriate sections before making your selection.

☒ Life sciences ☐ Behavioural & social sciences ☐ Ecological, evolutionary & environmental sciences

For a reference copy of the document with all sections, see nature.com/documents/nr-reporting-summary-flat.pdf

Life sciences study design

All studies must disclose on these points even when the disclosure is negative.

Sample size	The pathological cohorts and samples utilized in this study are unique. Consequently, every sample of sufficient quality was included in the study. Notably, controls were matched as closely as possible in terms of age, sex and pathology. In the case of the lecanemab sample, controls were selected based on the pathological resemblance and APOE4 genotype, albeit without prior exposure to the drug.
Data exclusions	Three of the 16 AD donors (cases 102-2, 102-3, and 102-9) were excluded due to low RNA quality, yielding a final cohort of 13 iAD samples for ST analysis. RNA quality was assessed based on DV200 scores, the number of genes detected per ST spot, and the percentage of mitochondrial genes per spot. For ST analysis, spots with extreme UMI counts, low feature counts, more than 20% mitochondrial expression (or over 30% for hippocampus), or those located at the slide edges or lacking tissue coverage were removed on a per-sample basis.<br><br>For scRNAseq, cells with mitochondrial gene expression above 20% and doublets were excluded. Additionally, AN1792 donor samples 102-7, 102-8, 102-11, and 102-21 were removed due to high contamination fractions, low UMI counts, or elevated mitochondrial expression.
Replication	There are no replications of the study cohort available. However, a subset of the samples was processed twice during a pilot study to test and set up ST, resulting in consistent data outputs.
Randomization	For AN1792 ST, each iAD sample was paired with a NND or NAD control sample when possible. Typically, 2–6 samples, often four, were processed concurrently on a staggered schedule. Almost all samples were sequenced simultaneously, with the exception of one iAD and four NND samples.<br><br>In lecanemab ST, frontal and temporal cortex samples from both the lecanemab-treated patient (NMA22-205) and an nAD control (NMA22-300) were processed and sequenced together. Parietal and hippocampal samples from both the lecanemab patient and the same nAD control were also co-processed and sequenced. Two additional nAD controls A14-193 and A11-170) were introduced later and were processed and sequenced together.<br><br>For the Visium HD processing, all samples were processed and sequenced together.<br><br>For single-cell RNA sequencing (scRNAseq), samples were processed and sequenced in batches. The first batch included four brain regions—frontal cortex, temporal cortex, parietal cortex, and hippocampus—from one nAD control (NMA22-205) and one lecanemab-treated sample (NMA22-300). The second batch included the same regions from two additional nAD controls (A14-193 and A11-170). The third batch consisted of frontal cortex samples from 10 AN1792 samples (102-1, 102-7, 102-8, 102-11, 102-15, 102-16, 102-17, 102-19, 102-21, and 102-22).
Blinding	During the ST workflow, it was not possible to identify the samples being used because the sample IDs were obscured by the cassette used in the ST process. For scRNAseq, the samples were coded to ensure unbiased analysis. Additionally, immunohistochemistry analyses were conducted by a researcher who was blinded to the group IDs.

Reporting for specific materials, systems and methods

We require information from authors about some types of materials, experimental systems and methods used in many studies. Here, indicate whether each material, system or method listed is relevant to your study. If you are not sure if a list item applies to your research, read the appropriate section before selecting a response.

Materials & experimental systems

n/a	Involved in the study	
☐	☒	Antibodies
☒	☐	Eukaryotic cell lines
☒	☐	Palaeontology and archaeology
☒	☐	Animals and other organisms
☒	☐	Clinical data
☒	☐	Dual use research of concern

Methods

n/a	Involved in the study	
☒	☐	ChIP-seq
☒	☐	Flow cytometry
☒	☐	MRI-based neuroimaging

Antibodies

Antibodies used	For immunofluorescent staining, primary antibodies were as follows: pan-amyloid-beta (Cell Signaling, clone D54D2, 8243, 1:1000), pan-amyloid-beta (Cell Signaling, clone D3D2N, 15126, 1:1000), pan-amyloid-beta (BioLegend, clone 4G8, 800708, 1:500), APOE (Sigma Aldrich, ab947, 1:500), CD68 (Abcam, clone KP1, ab955, 1:400), Iba1 (Abcam, ab5076, 1:150), Iba1 (WAKO, 019-19741, 1:400), TMS1/ASC (Abcam, clone RM1049, ab309497, 1:100), A2M (Abcam, clone EPR4432, ab109422, 1:250), APOC1 (Abcam, clone EPR16813, ab198288, 1:200), SPP1 (Abcam, ab8448, 1:100), and DAPI (ThermoFisher, 62248, 1:5000). Secondary antibodies were as follows: Donkey anti-Mouse 488 (Invitrogen, A32766, 1:400), Donkey anti-Rabbit 568 (Invitrogen, A10042, 1:400), Donkey anti-Goat 647 (Invitrogen, A21447, 1:400), Donkey anti-Goat 568 (Invitrogen, A11057, 1:400), Donkey anti-Rabbit 647 (Invitrogen, A31573, 1:400), Donkey anti-Rabbit 594 (Invitrogen, A21207, 1:400), Donkey anti-Mouse 647 (Invitrogen, A31571, 1:400), and Donkey anti-Goat 488 (Invitrogen, A11055, 1:400).
Validation	All antibodies are commercially available and have been tested in human samples.<br>Website reference:<br>pan-amyloid-beta (Cell Signaling, clone D3D2N, 15126, 1:1000) https://www.cellsignal.com/products/primary-antibodies/b-amyloid-d3d2n-mouse-mab/15126<br>pan-amyloid-beta (Cell Signaling, clone D54D2, 8243, 1:1000) https://www.cellsignal.com/products/primary-antibodies/b-amyloid-d54d2-xp-rabbit-mab/8243<br>pan-amyloid-beta (BioLegend, clone 4G8, 800708, 1:500)<br>APOE (Sigma Aldrich, ab947, 1:500) https://www.sigmaaldrich.com/US/en/product/mm/ab947<br>CD68 (Abcam, clone KP1, ab955, 1:400) https://www.abcam.com/products/primary-antibodies/cd68-antibody-kp1-ab955.html<br>Iba1 (Abcam, ab5076, 1:150) https://www.abcam.com/products/primary-antibodies/iba1-antibody-ab5076.html<br>Iba1 (WAKO, 019-19741, 1:400) https://labchem-wako.fujifilm.com/us/product/detail/W01W0101-1974.html<br>TMS1/ASC (Abcam, clone RM1049, ab309497, 1:100) https://www.abcam.com/en-us/products/primary-antibodies/tms1-asc-antibody-rm1049-ab309497<br>A2M (Abcam, clone EPR4432, ab109422, 1:250) https://www.abcam.com/en-us/products/primary-antibodies/alpha-2-macroglobulin-antibody-epr4432-ab109422<br>APOC1 (Abcam, clone EPR16813, ab198288, 1:200) https://www.abcam.com/en-us/products/primary-antibodies/apolipoprotein-ci-apo-ci-antibody-epr16813-ab198288<br>SPP1 (Abcam, ab8448, 1:100) https://www.abcam.com/en-us/products/primary-antibodies/osteopontin-antibody-ab8448<br>DAPI (ThermoFisher, 62248, 1:5000) https://www.thermofisher.com/order/catalog/product/62248<br>Donkey anti-Mouse 488 (Invitrogen, A32766, 1:400) https://www.thermofisher.com/antibody/product/Donkey-anti-Mouse-IgG-H-L-Highly-Cross-Adsorbed-Secondary-Antibody-Polyclonal/A32766<br>Donkey anti-Rabbit 568 (Invitrogen, A10042, 1:400) https://www.thermofisher.com/antibody/product/Donkey-anti-Rabbit-IgG-H-L-Highly-Cross-Adsorbed-Secondary-Antibody-Polyclonal/A10042<br>Donkey anti-Goat 647 (Invitrogen, A21447, 1:400) https://www.thermofisher.com/antibody/product/Donkey-anti-Goat-IgG-H-L-Cross-Adsorbed-Secondary-Antibody-Polyclonal/A-21447<br>Donkey anti-Goat 568 (Invitrogen, A11057, 1:400) https://www.thermofisher.com/antibody/product/Donkey-anti-Goat-IgG-H-L-Cross-Adsorbed-Secondary-Antibody-Polyclonal/A-11057<br>Donkey anti-Rabbit 647 (Invitrogen, A31573, 1:400) https://www.thermofisher.com/antibody/product/Donkey-anti-Rabbit-IgG-H-L-Highly-Cross-Adsorbed-Secondary-Antibody-Polyclonal/A-31573<br>Donkey anti-Rabbit 594 (Invitrogen, A21207, 1:400) https://www.thermofisher.com/antibody/product/Donkey-anti-Rabbit-IgG-H-L-Highly-Cross-Adsorbed-Secondary-Antibody-Polyclonal/A-21207<br>Donkey anti-Mouse 647 (Invitrogen, A31571, 1:400) https://www.thermofisher.com/antibody/product/Donkey-anti-Mouse-IgG-H-L-Highly-Cross-Adsorbed-Secondary-Antibody-Polyclonal/A-31571<br>Donkey anti-Goat 488 (Invitrogen, A11055, 1:400) https://www.thermofisher.com/antibody/product/Donkey-anti-Goat-IgG-H-L-Cross-Adsorbed-Secondary-Antibody-Polyclonal/A-11055
